# *De novo* generation of a bright blue fluorophore from 2-oxoglutarate in biological samples†

**DOI:** 10.1039/d1sc05808h

**Published:** 2021-12-07

**Authors:** Yumin Kim, Sangyoon Kang, Byung Hun Lee, Youngjun Song, Sunah Kang, Hye Yoon Park, Yan Lee

**Affiliations:** Department of Chemistry, College of Natural Sciences, Seoul National University Seoul 08826 Korea gacn@snu.ac.kr; Department of Physics and Astronomy, College of Natural Sciences, Seoul National University Seoul 08826 Korea hyeyoon.park@snu.ac.kr

## Abstract

We discovered the generation of a new bright blue fluorophore from a particular type of amine and 2-oxoglutarate (2-OG) under mild conditions without any chemical additives. Two β-aminoethylamine molecules and three 2-OG molecules form an unprecedented 2-pyridone structure with a fused γ-lactam ring (DTPP) *via* complex reactions including double decarboxylation and quintuple dehydration. The DTPP fluorophore shows a high quantum yield (80%) and photostability. The great potential of the present DTPP generation in the quantitative analysis of 2-OG in biosamples is demonstrated.

## Abstract 2

DTPP, a bright fluorophore with 2-pyridone and γ-lactam, is generated in a *de novo* manner from non-fluorogenic 2-oxoglutarate and specific amines. The DTPP-based fluorometry is applied to the analysis of 2-oxoglutarate in a biological sample.

## Graphical abstract

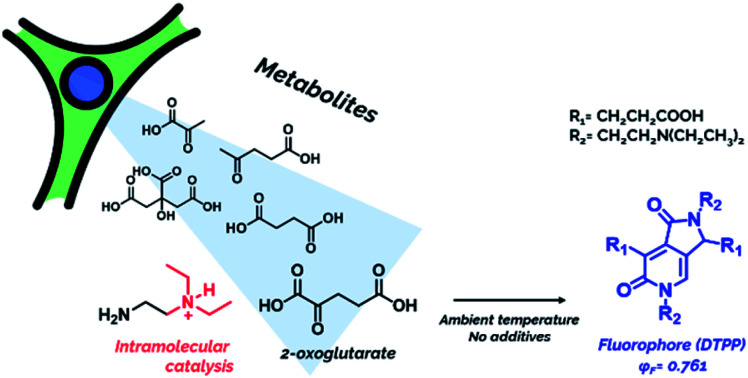

## Introduction

In biomedical applications, most chemical fluorophores are synthesized prior to their introduction into biological molecules, which can be thereby quantified or tracked for sensitive and selective analysis and imaging.^1,2^ In contrast, some biological fluorophores can be *de novo* generated from nonfluorogenic structures contained in biomolecules, as illustrated by the fluorophore maturation of green fluorescence protein from serine, tyrosine, and glycine residues.^3^ The *de novo* fluorophore generation in biological systems not only shed some light on the evolution of biopigments and the origin of autofluorescence but also inspired synthetic chemists to design next-generation fluorophores.^4–6^

Herein, we report a serendipitous discovery of a new bright blue fluorophore possessing a fused 2-pyridone ring, which is *de novo* generated from 2-oxoglutarate (2-OG) and primary amines without the need for additives or a heat source. Through a detailed analysis of the structure and properties of the new fluorophore, we propose a possible mechanism for the highly selective formation of the fluorophore on reactant structures. Furthermore, we suggest a quantitative method for the analysis of 2-OG, one of the key metabolites in the citric acid cycle, based on the direct generation of the fluorophore from 2-OG in biological samples.

## Results and discussion

**Scheme 1:**
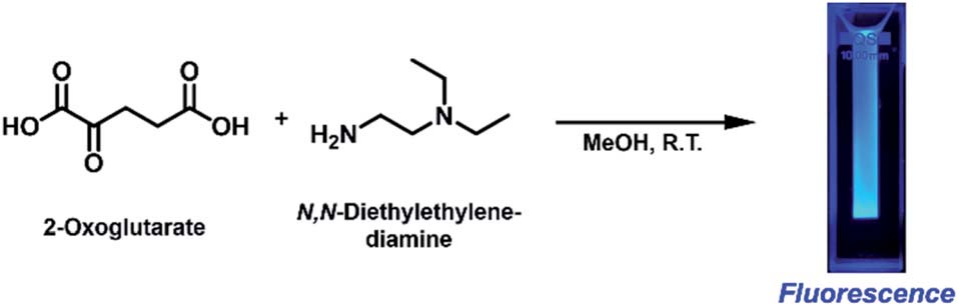
Fluorescence generation from 2-oxoglutarate (2-OG) and *N*,*N*-diethylethylenediamine (DEEDA) at ambient temperature without any additives.

We initially intended to form an imine bond between the α-carbonyl group of 2-OG and the primary amine group of *N*,*N*-diethylethylenediamine (DEEDA) by stirring in methanol (MeOH) at room temperature overnight (Scheme 1). A strong blue emission was unexpectedly observed in the reaction mixture upon 365 nm illumination with a hand-held UV lamp. The blue-emitting substance was very hydrophilic and strongly adsorbed on silica gel resins. The compound showed affinity for both cationic and anionic resins, which was indicative of its zwitterionic properties. Through a rigorous purification process using cationic and anionic exchange followed by reverse-phase chromatography (see the ESI† for details), we finally obtained a pure substance as a yellow viscous liquid (purification yield: 0.034% molar equivalent compared with the initial amount of 2-OG).

From the *m*/*z* value of the fluorescent substance, which was determined to be 493.30 (Fig. S5a†) from the liquid chromatography-mass spectroscopy (LC-MS) spectra, we inferred that it was generated by complex reactions between three 2-OG (146.10 g mol^−1^) molecules and two DEEDA (116.21 g mol^−1^) molecules, including double decarboxylation (–2CO_2_) and quintuple dehydration (–5H_2_O). This assumption was supported by high-resolution tandem mass spectrometry (HRMS-MS) analysis, in which the scanning for [M + H]^+^ allowed the detection of an *m*/*z* value of 493.3010, along with fragments with *m*/*z* values of 420.2109, 347.1231, 335.1228, 110.1118, 86.0968, and 72.0815 (Fig. S5b and c†), consistent with the generation of a compound with a molecular formula of C_25_H_40_N_4_O_6_.

**Fig. 1:**
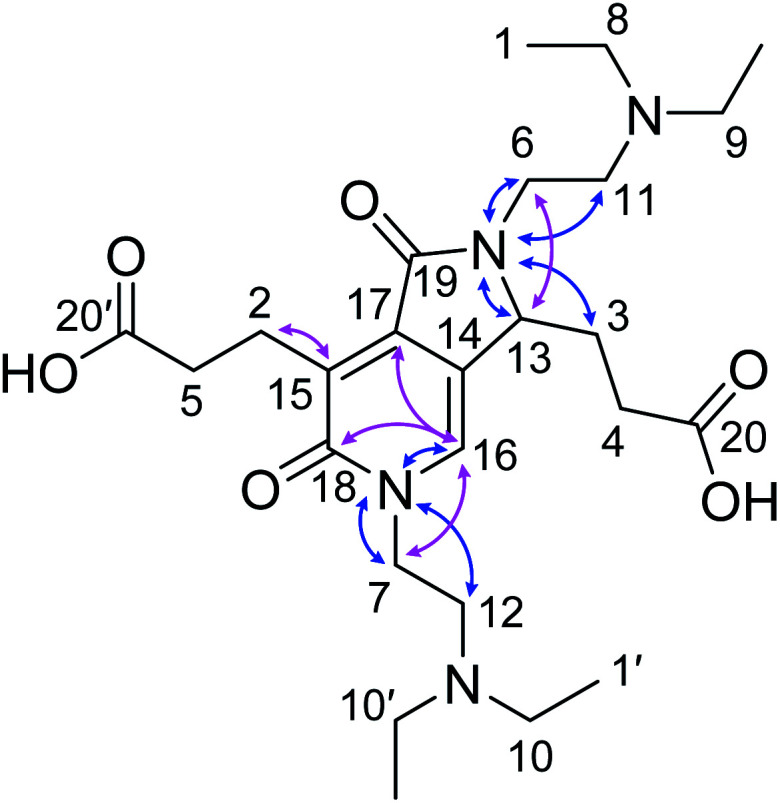
Identified structure of DTPP. Identified structure of DTPP. Representative HMBC and ^1^H–^15^N HMBC NMR correlations are indicated by pink and blue arrows, respectively. The labels of the carbon atoms correspond to those in Table 1.

From the ^1^H (Fig. S9†), ^1^H–^1^H correlation spectroscopy (COSY) (Fig. S11†), and ^1^H–^1^H nuclear Overhauser effect spectroscopy (NOESY) NMR spectra (Fig. S14†), the connectivity and spatial relationship between hydrogen atoms were elucidated. Similarly, the connectivity between carbon and hydrogen atoms was established by ^13^C (Fig. S10†), heteronuclear single quantum coherence (HSQC) (Fig. S12†), and heteronuclear multiple bond correlation (HMBC) NMR spectroscopy (Fig. S10†). Furthermore, a ^15^N–^1^H HMBC NMR spectrum (Fig. S13†) was recorded to obtain information on the connectivity between nitrogen and hydrogen atoms. Table 1 summarizes the correlation results. Taken together, these results confirmed the structure of the fluorescent compound as 3,3′-(2,5-bis(2-(diethylamino)ethyl)-1,6-dioxo-2,3,5,6-tetrahydro-1*H*-pyrrolo[3,4-*c*]pyridine-3,7-diyl)dipropionic acid (DTPP, 1), which consists of a 2-pyridone structure with a fused γ-lactam ring and dangling tertiary amines and carboxylic acids (Fig. 1). Representative HMBC and ^1^H–^15^N HMBC interactions are indicated with cyan and purple arrows, respectively. The MS-MS fragmentation pattern (Fig. S5b†) is in agreement with the loss of two DEEDA residues from DTPP. The DTPP structure was further supported by a single crystal X-ray diffraction analysis of the core structure (Fig. S27†).

**Table 1:** Important ^1^H, ^13^C, HSQC, HMBC, COSY, and NOESY NMR data for the identification of DTPP

C peaks at *δ*_C_ (ppm)	C–H correlation at *δ*_C_		H–H correlation at *δ*_H_^a^		Assignment of C atoms^b^
	*δ*_H_ (ppm) showing HSQC correlations (multiplicity, proton number)	*δ*_H_ (ppm) showing strong HMBC correlations	*δ*_H_ (ppm) showing ^1^H–^1^H COSY correlations	*δ*_H_ (ppm) showing strong NOESY correlation	
7.96–8.23	1.28–1.30 (m, 12H)	3.29–3.32	3.29–3.32	3.29–3.32, 3.40, 3.53	C1 and C1′
19.66	3.24–3.25 (m, 2H)	2.59	2.59	2.59	C2
25.05	2.32 (m, 2H)	1.96–2.05, 4.87	1.96–2.05, 4.87	1.96–2.05, 3.64, 4.87, 7.86	C3
26.78	1.96–2.05 (m, 2H)	2.32, 4.87	2.32	2.32	C4
32.33	2.59 (m, 2H)	3.24–3.25	3.24–3.25	3.24–3.25	C5
35.37	3.64, 4.16 (dt, 2H)	3.40	3.40	2.32,3.29–3.32, 3.40, 3.64, 4.16, 4.87	C6
46.55	4.44 (t, 2H)	3.53, 7.86	3.53	1.28-1.30,3.29-3.32, 3.53, 7.86	C7
47.32	3.29–3.32 (m, 2H)	3.40	1.28–1.30	Complicated	C8
48.07	3.29–3.32 (m, 2H)	3.40	1.28–1.30	Complicated	C9
48.26–48.27	3.29–3.32 (m, 4H)	3.53	1.28–1.30	Complicated	C10 and C10′
48.63	3.40 (t, 2H)	3.64, 4.16	3.64, 4.16	1.28–1.30, 4.87	C11
50.23	3.53 (t, 2H)	4.44	4.44	1.28–1.30	C12
56.54	4.87 (t, 1H)	1.96–2.05, 2.32, 3.64, 4.16, 7.86	2.32	3.64, 4.16	C13
120.90	—	2.32, 4.87, 7.86	—	—	C14
129.39	—	2.59, 3.24–3.25, 7.86	—	—	C15
131.86	7.86 (s, 1H)	4.44, 4.87	—	3.53, 4.44	C16
139.30	—	3.24–3.25, 7.86	—	—	C17
164.03	—	3.24–3.25, 4.44, 7.86	—	—	C18
168.08	—	3.64, 4.16, 7.86	—	—	C19
177.12	—	1.96–2.05, 2.32	—	—	C20
177.19	—	2.59, 3.24–3.25	—	—	C20′

a Correlation with the H peaks at *δ*_H_ ppm of HSQC.

b Carbon numbers are in the order of *δ*_C_ ppm and shown in Fig. 1.

**Fig. 2:**
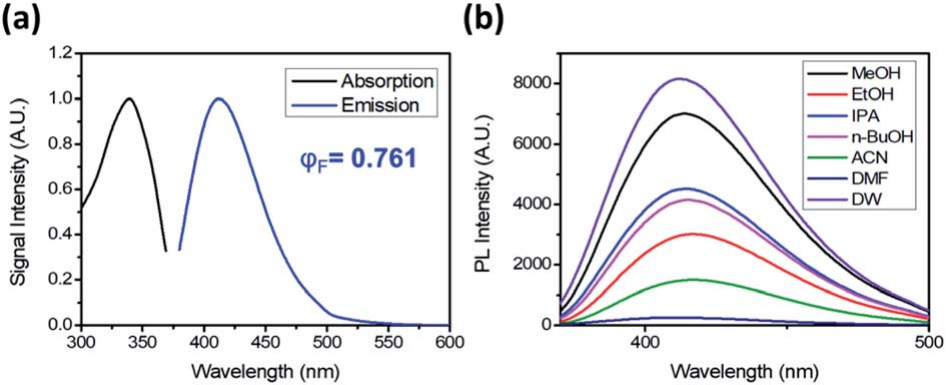
Photoluminescence of DTPP. (a) Absorption and emission spectra of DTPP in deionized water (DW). (b) Emission spectra of DTPP in various solvents. The emission spectra were obtained with excitation at 343 nm. The DTPP concentration was 40 µM.

DTPP has an absorption maximum at 340 nm (*ε* = 3470 L mol^−1^ cm^−1^) and an emission maximum at 413 nm in water (Fig. 2a). It shows a relatively small bathochromic shift (∼10 nm) but a large increase in fluorescence intensity as the solvent polarity increases (Fig. 2b and S1a†). The relative quantum yield (*φ*_F_) was calculated by using quinine sulfate as the reference and the emission spectra upon excitation at 354 nm (Fig. S1b†). The *φ*_F_ values in polar solvents such as water and methanol are approximately 75%, which are comparable to those of well-known fluorescent dyes such as fluorescein (*φ*_F_ ∼ 80%) and 4′,6-diamidino-2-phenylindole (*φ*_F_ ∼ 60%).^7,8^

**Fig. 3:**
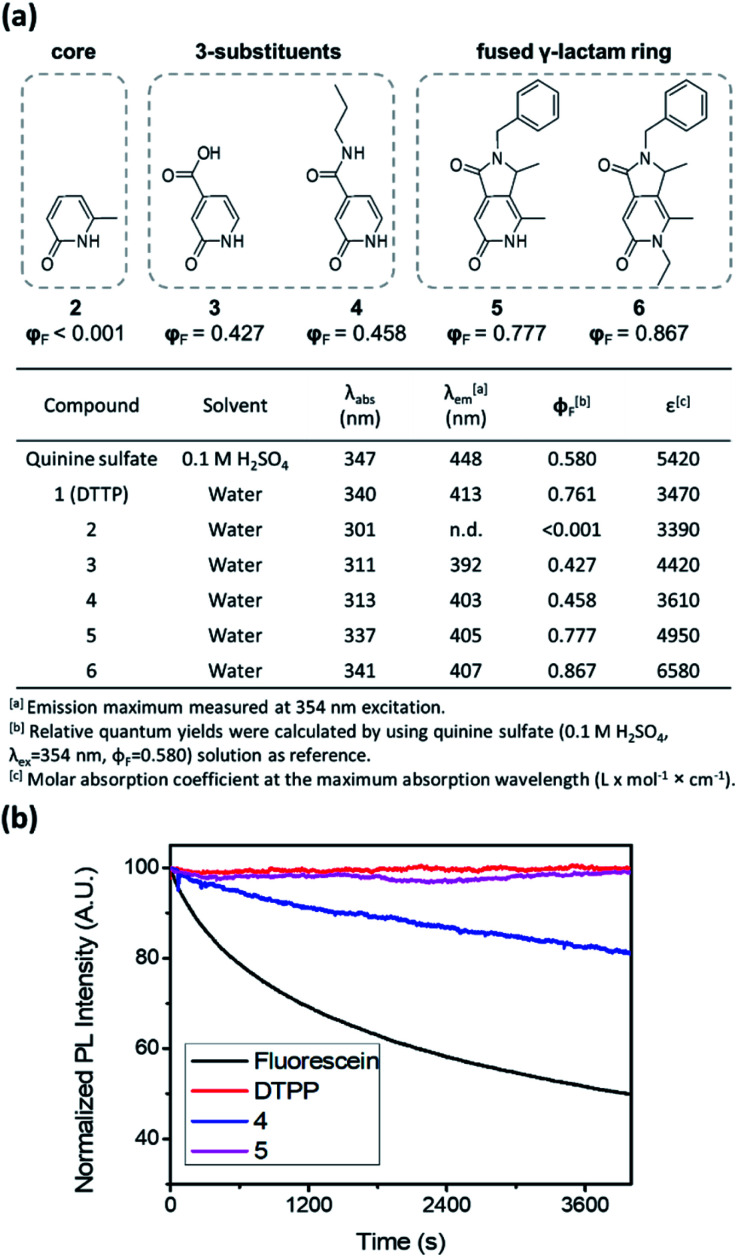
Structure–photophysical property relationships of the DTPP analogues. (a) Photophysical data summary of the DTPP analogues (1–6). (b) Photostability of DTPP, compounds 4 and 5, and fluorescein during irradiation with an Xe lamp.

To unveil the role of each structural moiety in DTPP in the generation of the strong blue fluorescence, we prepared a series of 2-pyridone analogues (2–6) and compared their fluorescence properties (Fig. 3a and S2†). Compound 2 having only the 2-pyridone ring has a significantly blue-shifted absorption maximum at 301 nm and a very low quantum yield (*φ*_F_ < 0.1%). However, upon introducing a carboxylic acid (3) or an amide group (4) at the 3-position of the 2-pyridone ring, the absorption maximum is shifted to 311–313 nm, and the quantum yield jumps to approximately 40–50%. The π-orbitals of the carboxylic acid and amide substituents may actively influence the HOMO–LUMO levels, thereby changing the fluorescence properties, as was similarly reported for other 2-pyridone-based fluorophores.^9,10^ In addition, we synthesized 2-pyridone derivatives with a fused γ-lactam ring (5 and 6) using other synthetic processes.^11^ Both compounds 5 and 6 show similar absorption and emission spectra to DTPP, and their quantum yields are enhanced up to 78–87%. The higher fluorescence quantum yields in the fused 2-pyridone structure are most likely due to the reduced nonradiative relaxation of excited electrons as a result of the lower rotational and vibrational modes of the lactam ring compared with those of the linear amide.^12^

The fused γ-lactam ring structure also significantly affects photostability. Irradiation with a Xe lamp (150 W) of compound 4 bearing a linear amide instead of the fused γ-lactam ring caused a gradual decrease in the fluorescence intensity, albeit at a slower rate than that of fluorescein (Fig. 3b). Meanwhile, compound 5 and DTPP maintained the fluorescence intensity upon irradiation for 100 min, indicating that the nonreversible deactivation of excited fluorophores can be effectively prevented by the stability of the fused γ-lactam ring.^13^

**Fig. 4:**
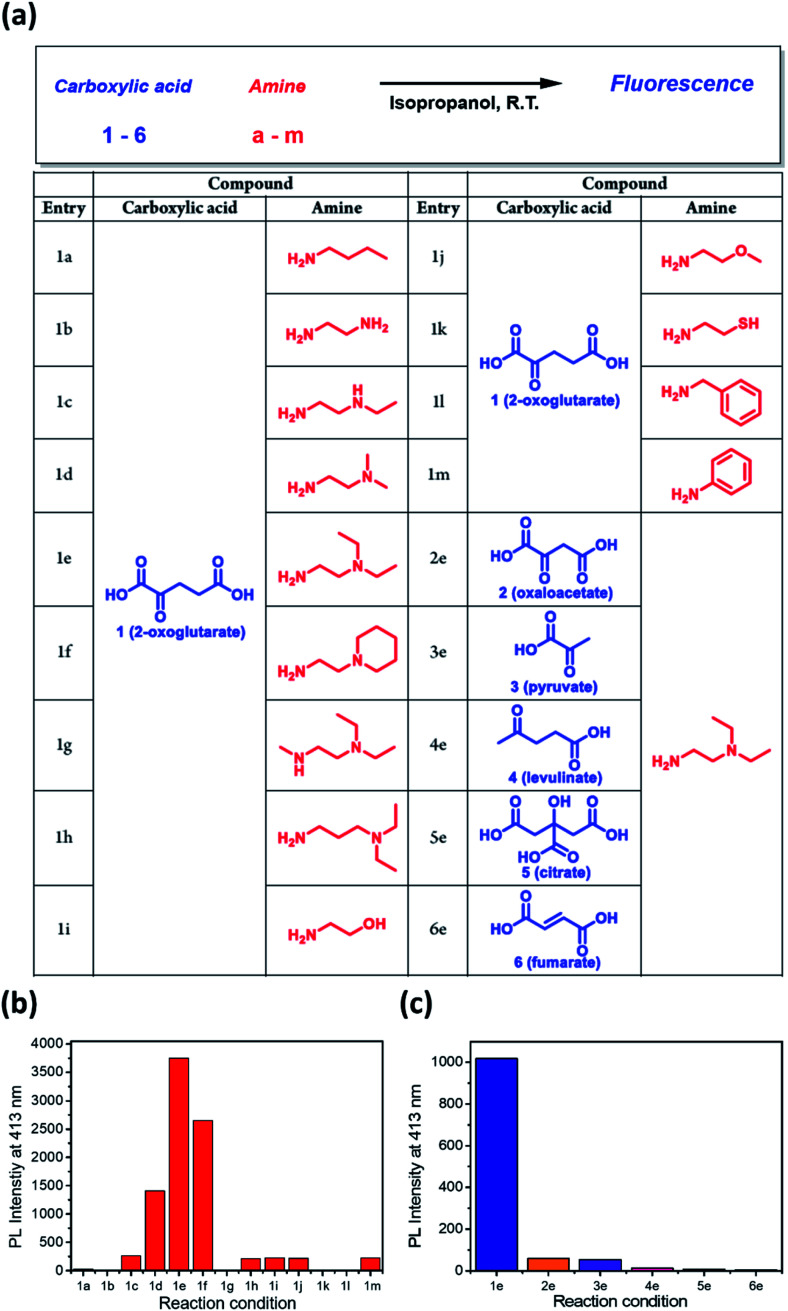
Selective generation of fluorescence from various amines and carboxylic analogues of 2-OG. (a) Structures of amines and carboxylates tested for fluorescence generation. (b) Relative fluorescence intensity of the reaction mixture between various amines and 2-OG. (c) Relative fluorescence intensity of the mixture between DEEDA (e) and various carboxylic analogues of 2-OG.

Since the dangling tertiary amines and carboxylic acids in the DTPP structure have no significant effect on either the quantum yield or photostability, we investigated whether similar fluorescent compounds could be generated from various amines and other carboxylic analogues of 2-OG by simple mixing and incubation (Fig. 4a). As shown in Fig. 4b, a simple aliphatic amine such as *n*-butylamine produced negligible fluorescence in a mixture with 2-OG (1a). Only 1,2-diamine compounds having primary and tertiary amine groups (1d, 1e, and 1f) afforded considerable fluorescence. Interestingly, 3-(diethylamino)propylamine, in which primary and tertiary amine groups are separated by a three-methylene spacer, showed much lower fluorescence intensity (1h). On the basis of the structure of DTPP, the primary amine can be considered critical for the formation of both 2-pyridone and γ-lactam rings. Additionally, the position of the tertiary amine seems to be essential for the efficient generation of the fluorophore.

The fluorophore formation is highly selective to 2-OG among other carboxylic analogues (Fig. 4c). The much lower fluorescence intensity of the mixture of DEEDA with pyruvate or levulinate indicates the importance of both carboxylic acid groups in 2-OG (3e and 4e). For the reaction mixture using citrate or fumarate, negligible fluorescence was observed, suggesting that the α-carbonyl group is essential for the fluorophore formation (5e and 6e). Furthermore, the limited fluorescence of the mixture using oxaloacetate reveals the importance of the 1,3-dicarboxylate groups (2e).

**Scheme 2:**
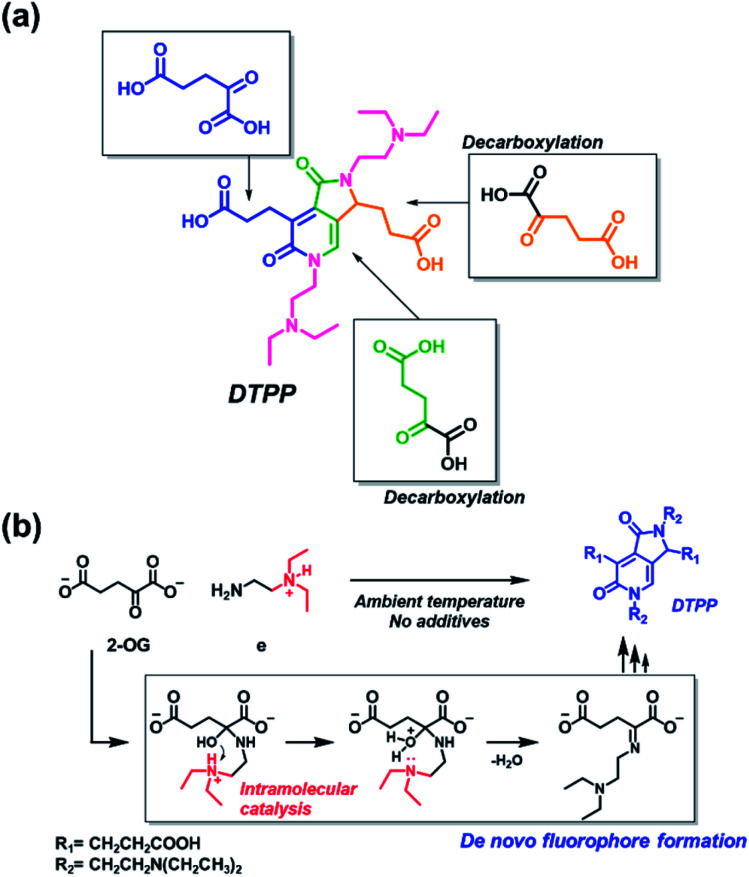
Generation of DTPP from two DEEDA and three 2-OG molecules. (a) Predicted origin of each atom in DTPP. The atom connectivity of DEEDA is indicated in magenta, and those of 2-OGs are indicated in blue, green, and orange in the DTPP structure. (b) Catalytic effect of the β-tertiary amine in DEEDA on the imine bond formation. The whole process for the DTPP formation is proposed in Fig. S3.†

The origin of each fragment in the DTPP structure can be predicted according to the connectivity of atoms in 2-OG and DEEDA, which is indicated with different colors in Scheme 2a. On the basis of the connectivity and selectivity results for the amine and carboxylic compounds described above, we propose a possible mechanism for the DTPP formation (Fig. S3†). The reaction might be initiated by the formation of an imine bond between 2-OG and DEEDA (step 1). The imine adduct would then attack a second 2-OG molecule *via* an aldol condensation reaction (steps 2 and 3), followed by decarboxylation of the product (step 4). After another aldol-like reaction with a third 2-OG molecule (step 5), a second DEEDA molecule might attack the product *via* a Michael-type reaction (step 6). Finally, the 2-pyridone and γ-lactam rings would be formed by internal condensation following decarboxylation (step 7). The *m*/*z* value for each predicted structure was detected in the LC-MS spectra of the reaction mixture. Table S1† summarizes the relative intensity of each MS peak. Notably, although the DTPP formation is a quite complex reaction among five substrates (three 2-OG molecules and two DEEDA molecules), it proceeds at room temperature in the absence of chemical additives.

The suggested mechanism could explain the importance of the β-tertiary amine in DEEDA during the fluorophore formation. In step 1, the protonated β-tertiary amine could act as an intramolecular acid catalyst donating a proton to the carbinol group, thereby facilitating the formation of the imine bond (Scheme 2b).^14^ We compared the tendency for imine formation using different amines, *i.e.*, DEEDA (e) possessing a β-tertiary amine or a γ-tertiary amine (h), according to LC-MS analysis. As a result, a much larger amount of the imine intermediate was formed from DEEDA compared with that obtained from h, supporting the catalytic effect of the β-tertiary amine in the imine formation (Fig. S4†). Moreover, the proposed mechanism also explains the selectivity for 2-OG, since the α-carbonyl group and the 1,3-dicarboxylate groups would be essential for the formation of the imine (step 1) and the 2-pyridone and γ-lactam rings (step 7), respectively.

Having established the origin of the bright blue fluorophore from DEEDA and 2-OG under mild conditions, a potential application of the fluorophore formation in biological samples could be suggested. 2-OG, also known as α-ketoglutarate, plays a key role in multiple metabolic and cellular pathways. As a main substrate in the anabolic and catabolic citric acid cycle, it regulates the amino acid synthesis, ATP production, and reductive/oxidative potential.^15–17^ In addition, the cytosolic level of 2-OG in the range 0.1–10 mM modulates the activity of 2-OG-dependent dioxygenases involved in the hypoxia-inducible factor response, DNA methylation, and histone modification controlling trained immunity and hematopoietic cell differentiation.^18–20^ Since the strong fluorescence is selectively generated only from 2-OG among various carboxylic acid metabolites (pyruvate, oxaloacetate, 2-OG, citrate, and fumarate) related to the citric acid cycle, we propose a selective fluorometric quantification method of 2-OG based on the *de novo* generation of DTPP.

**Fig. 5:**
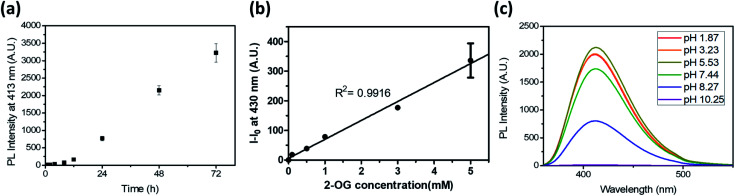
(a) Time-dependent fluorescence generation at 413 nm (*λ*_ex_ = 343 nm) of a 2-OG/DEEDA mixture (10 mM in MeOH). (b) Photoluminescence intensity of the reaction mixture in MeOH after 24 h according to the 2-OG concentration. The DEEDA concentration was fixed as 10 mM. The intensity was measured at 430 nm (*λ*_ex_ = 343 nm). (c) Emission spectra of DTPP at 20 µM in acetate, phosphate, and borate buffers under acidic, neutral, and basic pH conditions, respectively. Data are means (±S.D.).

To test the potential of the DTPP-based 2-OG quantification method, we first measured the kinetics of the DTPP formation (Fig. 5a). In a mixture of 2-OG (10 mM) and DEEDA (10 mM) in MeOH, the fluorescence intensity at 430 nm increased in a linear manner over time after an initiation time of about 4 h. The reaction somewhat slowed down after 51 h but it did not reach a steady state even after 192 h (Fig. S6†). Considering the photostability of DTPP, the result indicates that the DTPP formation had not reached the thermodynamic equilibrium yet. At a different concentration (0.5–3 mM) of 2-OG, the fluorescence intensity showed a linear increase according to the incubation time before 30 h (Fig. S7a–e†). Reversely, when we compared the fluorescence intensity at various concentrations of 2-OG at each time point, the fluorescence intensity showed a clear linear relationship with the 2-OG concentration (Fig. S7f†). Therefore, although the DTPP formation does not reach the equilibrium, we suppose that the DTPP fluorescence intensity can be a barometer of the substrates at a specific time point. Fig. 5b shows the linear relationship (*R*^2^ = 0.9916) between the fluorescence intensity and the 2-OG concentration after we incubated an excess amount of DEEDA (10 mM) with various amounts of 2-OG (100 µM–5 mM) in MeOH for 24 h at room temperature as a standard condition.

We also evaluated the pH dependency of the DTPP fluorescence. As shown in Fig. 5c, DTPP exhibited the maximum fluorescence intensity at pH 5–6, but a similar level of intensity was observed in the range of pH 1–7. Interestingly, the fluorescence was almost completely lost above pH 10. Since the pH level in most physiological fluids is in the range 4–8,^21^ the DTPP fluorescence intensity could be used to quantitatively estimate the 2-OG content in most biological samples.

**Fig. 6:**
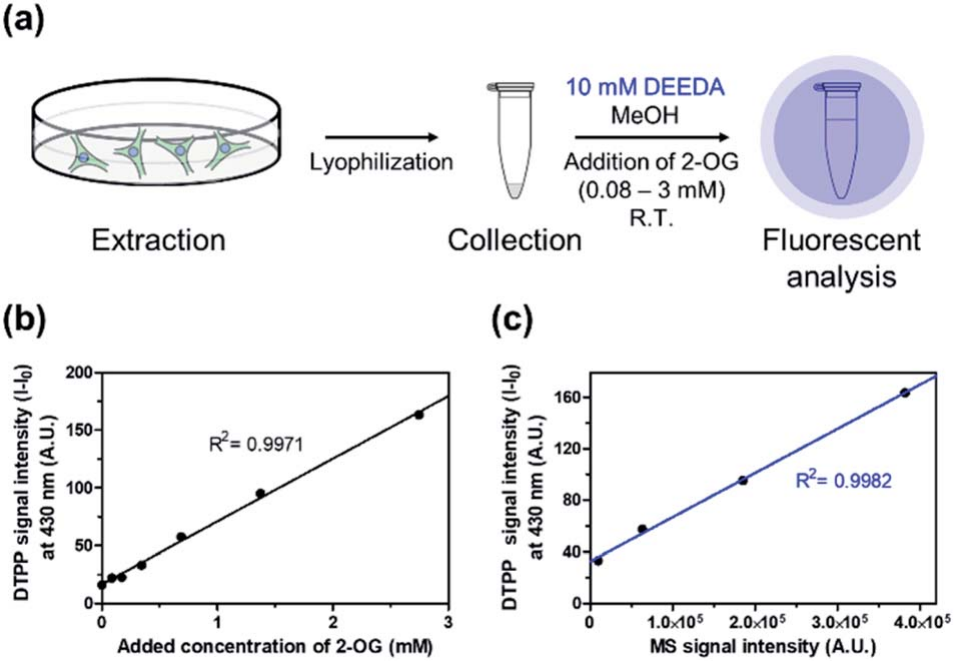
Verification of DTPP-based fluorometric analysis in biological samples. (a) Schematic diagram of the fluorometric analysis of 2-OG in cell extracts. The detailed protocol is described in the ESI.† (b) Fluorescence–concentration relationship in the DTPP method. The intensity was measured at 430 nm (*λ*_ex_ = 343 nm). (c) Linear relationship between the MS signal intensity from a conventional MS analysis (*x*-axis) and the fluorescence signal intensity from the DTPP method (*y*-axis) of the same samples. Data are means (±S.D.).

Then, we verified the potential of the DTPP fluorometric analysis in HeLa (human cervical cancer) cell extracts. Different amounts of 2-OG were added to extracts from 2.5 × 10^6^ HeLa cells (0.08–3 mM), and the resulting samples were incubated with 10 mM DEEDA at room temperature for 24 h (Fig. 6a). As can be seen in Fig. 6b, the incubated mixture showed a linear increase in the fluorescence intensity with the 2-OG concentration (*R*^2^ = 0.9971) and the limit of detection (LOD) was calculated to be 228.82 µM, which can be applicable to detect the variation of intracellular 2-OG concentration. High-resolution liquid chromatography-mass spectrometry (HRLC-MS) is the standard quantification method for the measurement of various metabolites including 2-OG.^22,23^ Accordingly, the cell extract samples containing 2-OG were analyzed by HRLC-MS, and the MS intensity signals were plotted against the fluorescence intensity obtained from the DTPP fluorometric method (Fig. 6c). A strict linear proportionality (*R*^2^ = 0.9982) was observed, supporting the potential of DTPP fluorometric analysis as the quantitative method in biological samples.

We also intended to measure the endogenous 2-OG level in the cells. In order to enhance the endogenous 2-OG concentration, HeLa cells were pre-treated with dimethyl 2-oxoglutarate (DM 2-OG), which can be converted to 2-OG by hydrolysis after permeation into the cell membrane.^24,25^ The cell extract was then treated with DEEDA to develop the DTPP fluorescence. As shown in Fig. S8,† the DM 2-OG-treated cells showed approximately two times higher fluorescence intensity than the non-treated control cells. The result clearly supported that our DTPP-based fluorescence assay could successfully detect the enhancement of the 2-OG concentration in cells.

**Fig. 7:**
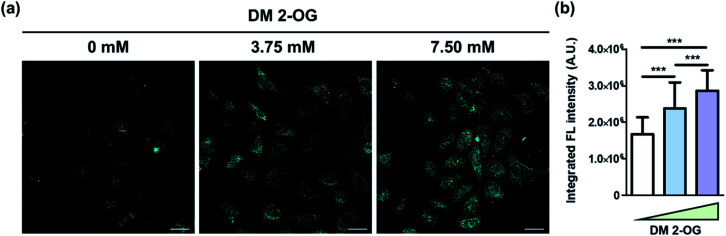
Visualization of DTPP generation in cells. (a) Representative images of HeLa cells pre-treated with various amounts of DM 2-OG for 24 h at 37 °C. The DTPP fluorescence was obtained by two-photon excitation at 720 nm and illustrated in cyan for a better view in the TPM images. Scale bar = 100 µm. Detailed protocol is described in the ESI.† (b) Averaged integrated fluorescence intensity of over 80 cells per group. Data are means (±S.D.). ***p < 0.001 as determined by Student's *t*-test.

Finally, we intended to show the *de novo* formation of DTPP from 2-OG and DEEDA can be used to visualize the relative amount of 2-OG in cells. Pre-existing methods to quantify the cellular 2-OG level generally loses the spatial information.^26,27^ However, we could develop the DTPP fluorescence in fixed cells without homogenization or excessive extraction. The two-photon microscopy (TPM) images taken with excitation at 720 nm clearly showed that the fluorescence intensity increased as the intracellular 2-OG level increased by the pre-incubation of DM 2-OG (Fig. 7a). Statistical analysis of over 80 cells supports the significant difference of the DTPP fluorescence among the cells treated with different concentrations of DM 2-OG (Fig. 7b).

On the basis of the results in quantification and bioimaging applications, we suggest that the *de novo* generation of the DTPP fluorophore, which is readily accomplished by adding DEEDA, can be a simple but effective method to detect 2-OG in biological samples.

## Conclusions

In summary, we discovered that a new blue fluorogenic structure, which was determined to be DTPP, is *de novo* generated from a β-tertiary amino ethylamine and 2-OG. A series of complex reactions leading to DTPP, including quintuple dehydration and double decarboxylation, proceed under mild conditions facilitated by the β-tertiary amine group, without requiring chemical additives. Both the 2-pyridone and fused γ-lactam rings are important for achieving a high quantum yield (∼80%) and DTPP photostability. The simple and quantitative DTPP formation has great potential in the fluorometric analysis of metabolites in biosamples. Furthermore, the discovery of the *de novo* generation of fluorophores from simple metabolites could help unveil the unknown origin of photoluminescence in biological systems.

## Data availability

All NMR, spectroscopic data, supplementary figures and tables, and detailed crystallographic information can be found in the ESI.† Crystallographic data for compound 6 has been deposited at the Cambridge Crystallographic Data Centre (CCDC): 2057122.

## Author contributions

Y. K. and S. K. contributed equally. Y. K. and S. K. conceived the project. Y. K., S. K., and Y. S. analyzed the NMR studies. S. K. synthesized the 2-pyridone analogues. Y. K. and S. K. designed and performed the spectroscopic analyses and SPPR studies. Y. K. and S. K. designed and performed fluorometric analysis in cellular extracts. Y. K. and B. H. L. designed and performed the fluorometric analysis in fixed cells. All authors contributed to prepare the manuscript and approved the final version of the manuscript. H. Y. P. and Y. L. supervised the whole project.

## Conflicts of interest

There are no conflicts to declare.

## Supplementary Material

SC-013-D1SC05808H-s001

SC-013-D1SC05808H-s002
