# Supplementary material for: De novo generation of a bright blue fluorophore from 2-oxoglutarate in biological samples
Source: Chem Sci. 2021 Dec 7;13(2):365–72. doi: 10.1039/d1sc05808h (PMC8729799; doi:10.1039/d1sc05808h)
Supplement: SC-013-D1SC05808H-s001 [file SC-013-D1SC05808H-s001.pdf]

Electronic Supplementary Material (ESI) for Chemical Science.
This journal is © The Royal Society of Chemistry 2021

Supporting Information

De Novo Generation of a Bright Blue Fluorophore from 2-Oxoglutarate in Biological Samples

Yumin Kim[†,1], Sangyoon Kang[†,1], Byung Hun Lee[2], Youngjun Song[1], Sunah Kang[1], Hye Yoon Park[*,2], and Yan Lee[*,1]

Affiliation:

[1]Department of Chemistry, College of Natural Sciences, Seoul National University, Seoul 08826, Korea

[2]Department of Physics and Astronomy, College of Natural Sciences, Seoul National University, Seoul 08826, Korea

**Corresponding Author E-mail:* gacn@snu.ac.kr *and hyeyoon.park@snu.ac.kr*

[†]These authors contributed equally to this work.

Table of contents

1. **Experimental section**

    1. 1. General methods

    1. 2. Synthesis

    1. 3. Quantum yield measurement

    1. 4. Photostability test

    1. 5. The cell culture

    1. 6. Fluorometric analysis of 2-OG in cell extracts

    1. 7. TPM analysis of 2-OG in fixed cells

2. **Photoluminescence data**
3. **A proposed mechanism of the DTTP generation**
4. **MS data**
5. **DTPP-based fluorometric analysis**
6. **NMR data**
7. **SC-XRD analysis**
8. **References**

1. Experimental section

1. 1. General methods

All reactions were performed in oven-dried glassware. 2-Oxoglutarate (2-OG), oxaloacetate, pyruvate, levulinate, citrate, fumarate, dimethyl 2-oxoglutarate (DM 2-OG), *N*-ethylenediamine (compound **c**), *N,N*-dimethylethylenediamine (compound **d**), *N,N*-diethylethylenediamine (DEEDA, compound **e**), 1-(2-aminoethyl)piperidine (compound **f**), *N,N*-diethyl-*N*′-methylethylenediamine (compound **g**), *N,N*-diethylpropylenediamine (compound **h**), benzylamine (compound **m**), acetylacetone, maleimide, ethylmaleimide, 2-hydroxyl-6-methylpyridine (compound **2**) and 2-hydroxyisonicotinic acid (compound **3**) were purchased from Tokyo Chemical Industry (Japan). Dicyclohexylcarbodiimide (DCC), methanol (MeOH), ethanol (EtOH), *n*-butanol, *N,N*-dimethylformamide (DMF), *n*-butylamine (compound **a**), Ethylenediamine (compound **b**), ethanolamine (compound **i**). 2-methoxyethylamine (compound **j**), 2-aminoethanethol (compound **k**) and aniline (compound **l**) were purchased from Sigma-Aldrich (USA). Propylamine was purchased from Junsei chemical (Japan). Triethylamine (TEA), acetonitrile (ACN) and dichloromethane (DCM) were purchased from Samchun (Korea). $CDCl_3$, D_2O and CD_3OD were purchased from BK instruments (Korea). Dulbecco's modified Eagle's medium (DMEM), Dulbecco's phosphate-buffered saline (DPBS), and trypsin-EDTA were purchased from Welgene (Gyeongsan, Korea). All chemical reagents and solvents were used without further purification. Thin layer chromatography was performed on glass coated with Silica gel 60 F254 and Silica gel 60 RP-18 F254s (Merck). Flash column chromatography was performed using silica gel (0.040-0.063 mm, Merck). Anion exchange column was performed using DOWEX® 1×8 chloride form (100-200 mesh, Sigma-Aldrich). Cation exchange column was performed using CM Sephadex® C-25 (Sigma-Aldrich). Proton NMR spectra were recorded at 300 MHz, 500 MHz or 850 MHz and chemical shifts were reported relative to the residual solvent peaks (for spectra recorded in $CDCl_3$; 7.26 ppm, D_2O; 4.79 ppm, CD_3OD; 3.31 ppm). Multiplicity was indicated as follow: s (singlet), d (doublet), t (triplet), q (quartet), h (hextet) and m (multiplet). Carbon NMR spectra were recorded at 75 MHz, 126 MHz or 214 MHz. Two-dimensional NMR spectra were recorded at 500 MHz or 850 MHz. NMR data were processed with MestReNova (Version 6.0.2) or Topspin (Version 4.0.6). High resolution MS data was recorded on the AB SCIEX Q-TOF 5600 LC/MSMS spectrometer using electrospray and atmospheric pressure chemical ionization. High performance liquid chromatography (HPLC) was performed on the Shimadzu

HPLC system equipped with an LC-20AD pump and an SPD-20A UV detector. Agilent ZORBAX 300SB-C18 5 µm 9.4 × 250 mm column was used for purification. For the mobile phase, Solution A (water with 0.1% v/v TFA) and Solution B (ACN with 0.1% v/v TFA) were used as eluents. FTIR spectra was recorded on the Thermo Scientific NICOLET iS10 spectrometer. UV absorption was recorded on the JASCO V-650 spectrophotometer and fluorescent emission was recorded on the JASCO FP-8300 spectrofluorometer.

1. 2. Synthesis

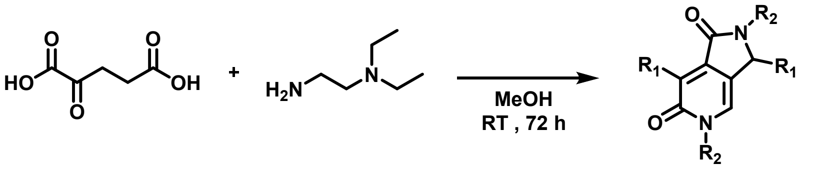

General procedure for the formation of 3,3'-(2,5-bis(2-(diethylamino)ethyl)-1,6-Dioxo-2,3,5,6-Tetrahydro-1H-Pyrrolo[3,4-c]Pyridine-3,7-diyl)dipropionic acid (DTPP, 1)

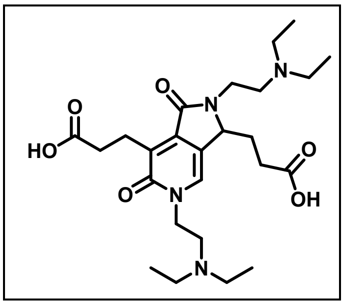

Generally, 2-OG or other carboxylic analogues (100 mM) were treated with DEEDA or various amine compounds (100 mM) in in MeOH, and the mixture was stirred at room temperature for overnight. Specifically, 2-OG (25.0 mmol, 3.65 g) was treated with DEEDA (25.0 mmol, 3.77 mL) in 250 mL of MeOH, and the mixture was stirred at room temperature for overnight. Then the solvent was removed under reduced pressure, and the residue was purified by cation exchange chromatography using an elution solution (water with 25% v/v ACN) and a washing solution (the elution solution with 0.125% v/v ammonium hydroxide). Then, the washed crude mixture was purified by anion exchange chromatography using an elution solution (water with 25% v/v ACN) and water solution (the elution solution with 0.572% v/v acetic acid). Finally, the washed compound was purified by HPLC eluted with 0-10% Solution B. Residual solvent was removed by lyophilization to afford a viscous yellow liquid (4.18 mg, yield 0.0342 %). ^{1}H NMR (850 MHz, D_2O) δ 7.86 (s, 1H), 4.87 (t, J = 3.6 Hz, 1H), 4.44 (t, J = 6.7 Hz, 2H), 4.16 (dt, J = 15.0, 7.5 Hz, 1H), 3.64 (dt, J = 15.1, 6.6 Hz, 1H), 3.53 (t, J = 6.6 Hz, 2H), 3.40 (t, J = 6.8 Hz, 2H), 3.32-3.24 (m, 10H), 2.59 (m, 2H), 2.39-2.31 (m, 2H), 2.10-1.98 (m, 1H), 1.30-1.28 (m, 12H). ^{13}C NMR (214 MHz, D2O) δ 177.19, 177.12, 168.08, 164.03, 139.30, 131.86, 129.39, 120.90, 56.54, 50.23, 48.63, 48.28, 48.26, 48.07, 47.32, 46.55, 35.37, 32.33, 26.78, 25.05, 19.66, 8.23, 8.13, 8.03, 7.96. MS(ESI)$^{+}$ calculated for $C_{25}H_{41}N_4O_6$ $[M+H]^{+}$: *m/z* 493.30, found 493.30.

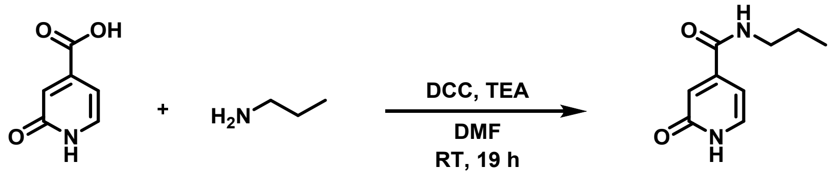

2-Oxo-N-propyl-1,2-dihydropyridine-4-carboxamide (4)

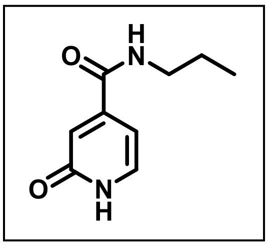

2-Hydroxyisonicotinicacid (100 mg, 0.719 mmol) was dissolved in 2 mL of DMF containing DCC (227 mg, 1.10 mmol) and TEA (99.0 µL, 0.714 mmol). The mixture was stirred for 30 min at rt. Propylamine (296 µL, 3.61 mmol) was added to the solution and stirred for 20 h at ambient temperature. The crude mixture was purified with DW/DCM extraction. The aqueous layer was washed with DCM several times and lyophilized. The product was further purified by HPLC using 0-13.5% Solution B to obtain white powder (56.2 mg, yield 43%). ^{1}H NMR (500 MHz, D_2O) δ 8.67 (s, 1H), 7.61 (d, J = 6.7 Hz, 1H), 6.84 (s, 1H), 6.72 (d, J = 6.7 Hz, 1H), 3.30 (t, J = 6.6 Hz, 2H), 1.58 (h, J = 7.4 Hz, 2H), 0.90 (t, J = 7.4 Hz, 3H). ^{13}C NMR (126 MHz, D_2O) δ 167.77, 164.52, 148.37, 135.86, 117.32, 106.23, 41.71, 21.61, 10.51. MS(ESI)$^{+}$ calculated for $C_9H_{13}N_2O_2$ [M+H]$^{+}$: *m/z* 181.10, found 181.10.

Synthetic procedure for compound 5 and 6

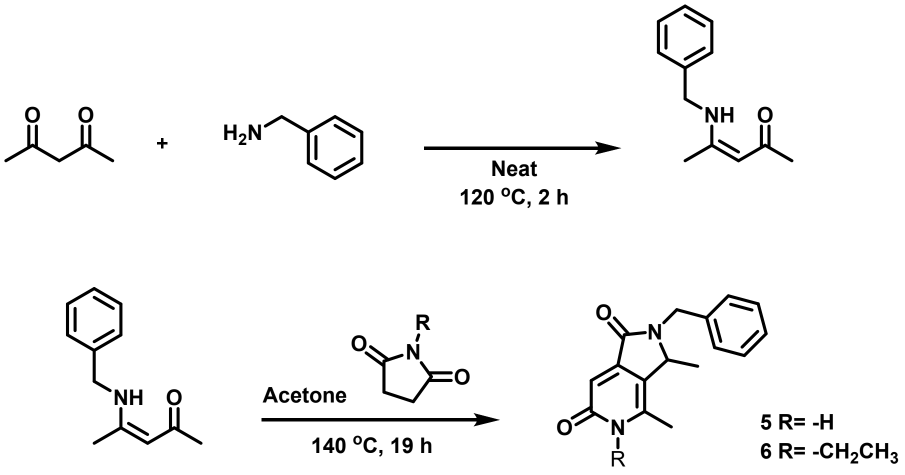

2-(Benzylamino)prop-l-enyl methyl ketone

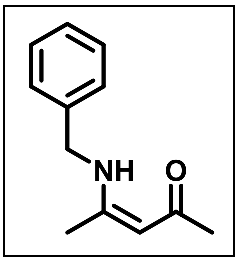

2-(Benzylamino)prop-l-enyl methyl ketone was obtained using previous reported synthetic methods [1]. Benzylamine (500 μL, 4.57 mmol) was added to acetylacetone (467 μL, 4.57 mol) without any solvent. After the mixture had been allowed to stir at 120 °C for 2 h, the mixture was purified by silica column chromatography with 2:1 hexane/ethyl acetate to afford yellow liquid (837 mg, yield 97.0 %). ^{1}H NMR (300 MHz, $CDCl_3$) δ 11.21 (s, 1H), 7.37 – 7.29 (m, 2H), 7.25 (d, J = 7.3 Hz, 3H), 5.06 (s, 1H), 4.40 (d, J = 6.3 Hz, 2H), 2.04 (s, 3H), 1.88 (s, 3H). ^{13}C NMR (126 MHz, $CDCl_3$) δ 194.81, 162.84, 137.77, 128.49, 127.10, 126.39, 95.59, 46.35, 28.59, 18.53. MS(ESI)$^+$ calculated for $C_{12}H_{17}NO$ $[M+H]^+$: *m/z* 190.12, found 190.11.

2-Benzyl-3,4-Dimethyl-3,5-Dihydro-1H-Pyrrolo[3,4-c]pyridine-1,6(2H)-Dione (5)

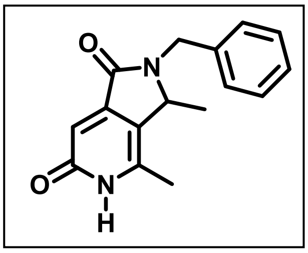

2-(Benzylamino)prop-l-enyl methyl ketone (588 μL, 3.19 mmol) was treated with maleimide (310 mg, 3.19 mol) in 16 mL of acetone. The mixture was refluxed for 24 h and acetone was removed under reduced pressure to afford a brown syrup. After acetone was removed under reduced pressure, the crude mixture was purified by petroleum ether precipitation. The supernatant was obtained and the residual solvent was removed under reduced pressure. Recrystallization with ethanol was performed to afford pale yellowish solid (520 mg, yield

61.0%). ^{1}H NMR (300 MHz, $CDCl_3$) δ 13.59 (s, 1H), 7.42 – 7.24 (m, 5H), 6.88 (s, 1H), 5.35 (d, *J* = 15.1 Hz, 1H), 4.32 (q, *J* = 6.4 Hz, 1H), 4.19 (d, *J* = 15.1 Hz, 1H), 2.40 (s, 3H), 1.43 (d, J = 6.4 Hz, 3H). ^{13}C NMR (126 MHz, $CDCl_3$) δ 166.21, 165.14, 145.51, 141.06, 136.08, 129.80, 128.90, 128.14, 128.01, 127.89, 118.72, 110.98, 52.88, 43.93, 17.69, 17.31, 16.31. MS(ESI)$^+$ calculated for $C_{16}H_{17}N_2O_2$ [M+H]$^+$: *m/z* 269.13, found 269.10.

2-Benzyl-5-Ethyl-3,4-Dimethyl-3,5-Dihydro-1H-Pyrrolo[3,4-c]pyridine-1,6(2H)-Dione (6)

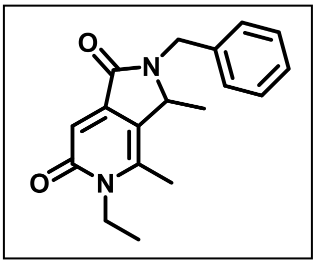

2-(Benzylamino)prop-l-enyl methyl ketone (588 μL, 3.19 mmol) was treated with ethylmaleimide (400 mg, 3.19 mol) in 32 mL of acetone. The mixture was refluxed for 24 h and the solvent was removed under reduced pressure to afford a brown syrup. The crude mixture was dissolved in 10 mL of *tert*-butyl alcohol. The solution was treated with *tert*-butoxide (260 mg, 2.31 mmol) and the mixture was refluxed at 120 °C for 21 h. After the solvent was removed under reduced pressure, the residual products were dissolved in cold water and acidified with acetic acid. The precipitated pale-yellow solid was filtered and further purified with silica gel column chromatography using 3:1 ethyl acetate/acetone to afford yellow solid (38.2 mg, yield 7%). ^{1}H NMR (500 MHz, CD_3OD) δ 7.38 – 7.27 (m, 5H), 6.70 (s, 1H), 5.14 (d, *J* = 15.2 Hz, 1H), 4.47 (q, *J* = 6.4 Hz, 1H), 4.41 (d, *J* = 15.2 Hz, 1H), 3.60 (q, *J* = 7.0 Hz, 2H), 2.33 (s, 3H), 1.43 (d, *J* = 6.5 Hz, 3H), 1.18 (t, J = 7.0 Hz, 3H). ^{13}C NMR (214 MHz, CD_3OD) δ 167.04, 165.55, 144.26, 144.05, 137.64, 129.96, 129.09, 128.93, 122.53, 110.85, 55.80, 44.99, 41.40, 18.40, 16.75, 13.58. MS(ESI)$^+$ calculated for $C_{18}H_{21}N_2O_2$ [M+H]$^+$: *m/z* 297.16, found 297.12.

1. 3. Quantum yield measurement

The relative quantum yields (φ_F) of each fluorophore was measured in reference to quinine sulfate (φ_F= 58% at 354 nm excitation in 0.1 M H_2SO_4). The formula used for measurements is shown below:

$$(\varphi_F)_{sample} = (\varphi_F)_{ref} \times [\frac{\left(\frac{PL_{area}}{OD}\right)_{sample}}{\left(\frac{PL_{area}}{OD}\right)_{ref}}] \times \eta^2{}_{sample}/\eta^2{}_{ref} \qquad (1)$$

where η is the refractive index of the solvent, and PL area and OD are the fluorescence area and absorbance value, respectively.

1. 4. Photostability test

Fluorescein (1.0 μM), DTPP (1.0 μM) and **4** (1.0 μM) in distilled water and **5** (1.0 μM) in EtOH were exposed to the light from a Xenon arc lamp (150W, UXL-159) in JASCO FP-8300 spectrofluorometer. Each sample was irradiated at the corresponding maximum absorption wavelength. Fluorescence emission spectra were measured continuously through 1 second intervals during the light exposure by using the time course measurement mode.

1. 5. The cell culture

HeLa (human cervical cancer) cells were grown in DMEM supplemented with 10% FBS at 37°C. The cells were seeded in a 100 mm culture dish at a density of 300,000 cells per dish. The cells were maintained at 37°C and 5% CO_2 for 72 h before further experiments. For enhancing the intracellular 2-OG concentration, the cells were incubated with 3.75 mM or 7.50 mM of DM 2-OG in DMEM supplemented with 10% FBS for 4 h at 37 °C before further experiments.

1. 6. Fluorometic analysis of 2-OG in cell extracts

Cell extracts were prepared according to the previous study [1]. Dishes with *HeLa* cells were taken out of the 37°C incubator and immediately placed on ice. Then, the growth medium was removed and cells were washed with 5 mL of deionized water. Subsequently, 5 mL of an extraction solvent (80% MeOH/H_2O), pre-cooled in an -80°C deep freezer for at least 1 h, was added to the cell culture dishes. The dishes were left for 15 min, and then cells were scraped with the extraction solvent at 4 °C. The scraped mixture was transferred to an Eppendorf tube and centrifuged at 14,000 rpm at 4 °C for 10 min. The supernatant was lyophilized and the dried cell extract was stored at -80°C before use.

For the quantitative analysis of 2-OG, the dried cell extract was dissolved to 1 mL of MeOH. Then, various amount of 2-OG was added to the mixture. For the fluorometric analysis, 1.4 µL (0.010 mmol) of DEEDA was added to the mixture and stirred at ambient temperature for 24 h. Fluorescent emission of each mixture at 430 nm was recorded on the JASCO FP-8300 spectrofluorometer excited at 343 nm. For comparison, the HRLC-MS intensity of 2-OG in the 2-OG-added cell extract was gained on 145.010-145.0200 m/z.

1. 7. Two-photon microscopy (TPM) analysis of 2-OG in fixed cells

HeLa (human cervical cancer) cells were grown in DMEM supplemented with 10% FBS at 37°C. The cells were seeded on 10 mm coverglasses in 6-well plates at a density of 150,000 cells per well. After incubating for 48 h, the growth medium was changed with fresh DMEM supplemented with 10% FBS containing 0 mM, 3.75 mM, or 7.50 mM of DM 2-OG. After incubation for 24 h at 37°C, the medium was removed and the cells were washed with 10 mL of PBS. Then, 10 mM DEEDA in isopropyl alcohol (5 mL) was added to each well. The DTPP fluorescence was developed by a further incubation for 24 h at 37°C. After washing the cells with DPBS twice, image acquisition was performed using a two-photon laser scanning microscope (FVMPE-RS; Olympus, Japan) with a 25× objective (XLPLN25XSVMP2; Olympus, Japan) and the Olympus Fluoview FV31S-SW Software. Fluorescence images of the DTTP distribution were acquired in the blue channel (λ_{ex} = 720 nm and λ_{em} = 410-455 nm).

All images were analyzed using the ImageJ software (National Institutes of Health, http://rsb.info.nih.gov/ij/). The total blue fluorescence intensity was integrated in each cell. The fluorescence intensity of more than 80 cells were analyzed per each group ($n > 80$).

2. Photoluminescence data

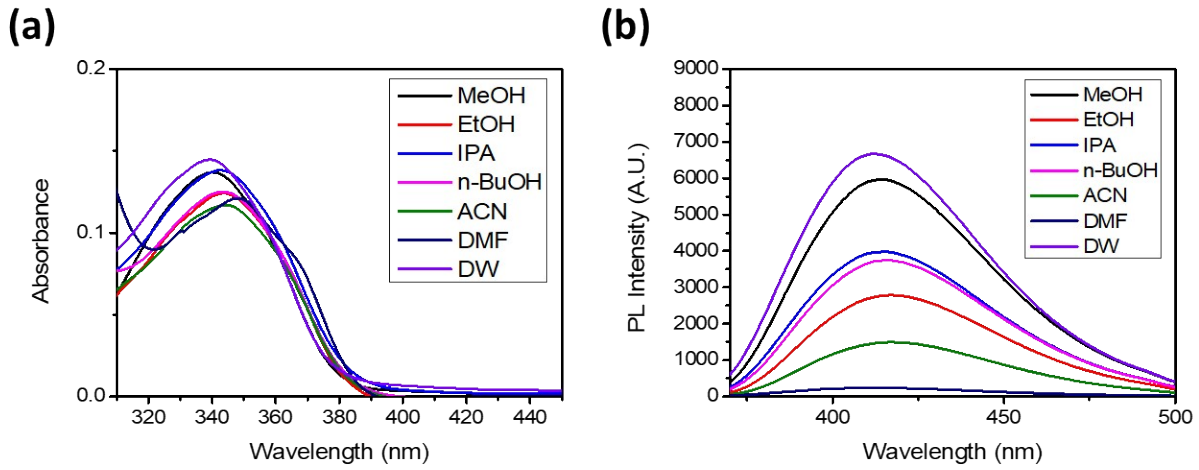

Figure S1. Absorption (a) and emission (b) spectra of DTTP (40 μM) in various solvents (methanol (MeOH), ethanol (EtOH), isopropanol (IPA), n-butyl alcohol (*n*-BuOH), acetonitrile (ACN), *N*,*N*-dimetylformamide (DMF), deionized water (DW). The emission spectra were obtained by the excitation at 354 nm.

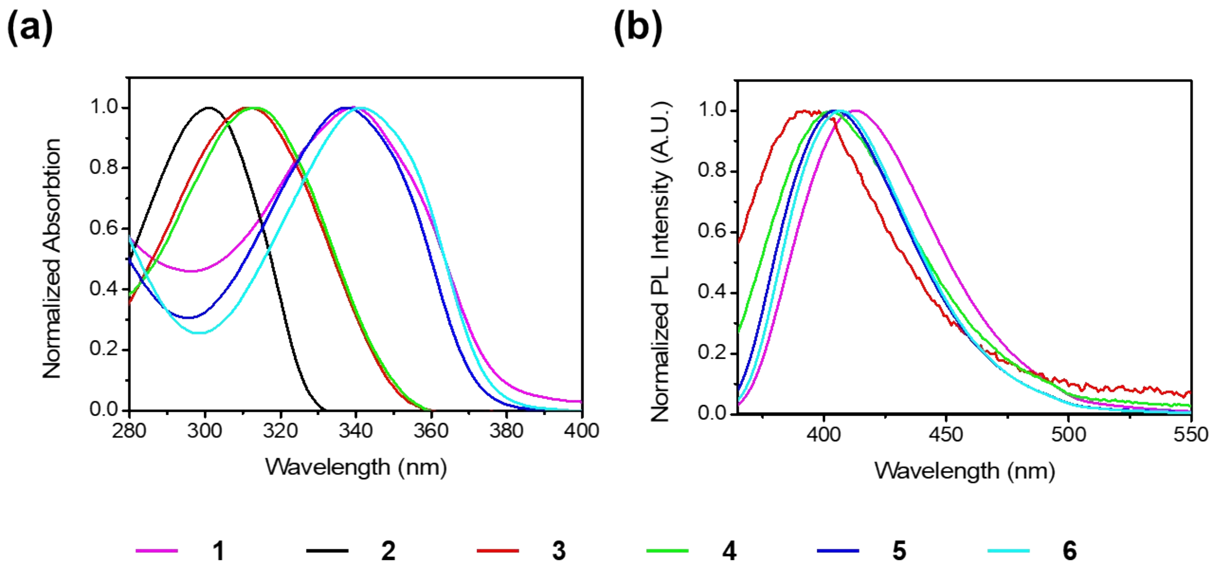

Figure S2. Normalized absorption (a) and emission (b) spectra of **1-6** in deionized water.

3. A proposed mechanism of the DTTP generation

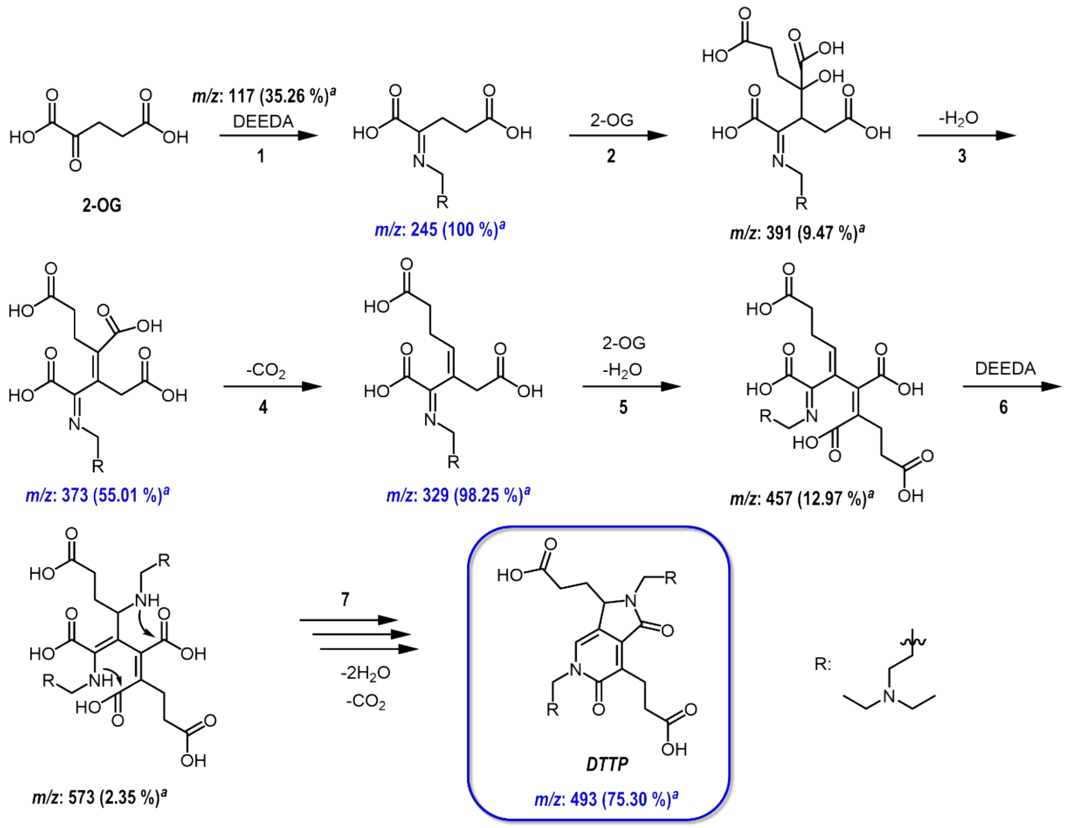

Figure S3. A proposed mechanism of DTPP generation from 2-OG and DEEDA. [a]Relative total ion currents (TIC) intensities in LC-MS (full-mass scanning). The TIC intensity of the imine intermediate (the 2nd product in the scheme, *m/z* = 245) was used as the reference (100%).

4. MS data

Table S1. The intensity of each peak in the reaction mixture. [a]Relative total ion currents (TIC) intensities in LC-MS (full-mass scanning). The TIC intensity of the imine intermediate (the 2nd product in the scheme, *m/z* = 245) was used as the reference (100%).

m/z	**Intensity**	**[a]Relative intensity (%)**
245	329779	100
391	31236	9.47
373	181405	55.01
329	324026	98.25
457	42799	12.97
573	7778	2.35
493	248325	75.30
117	116301	35.26

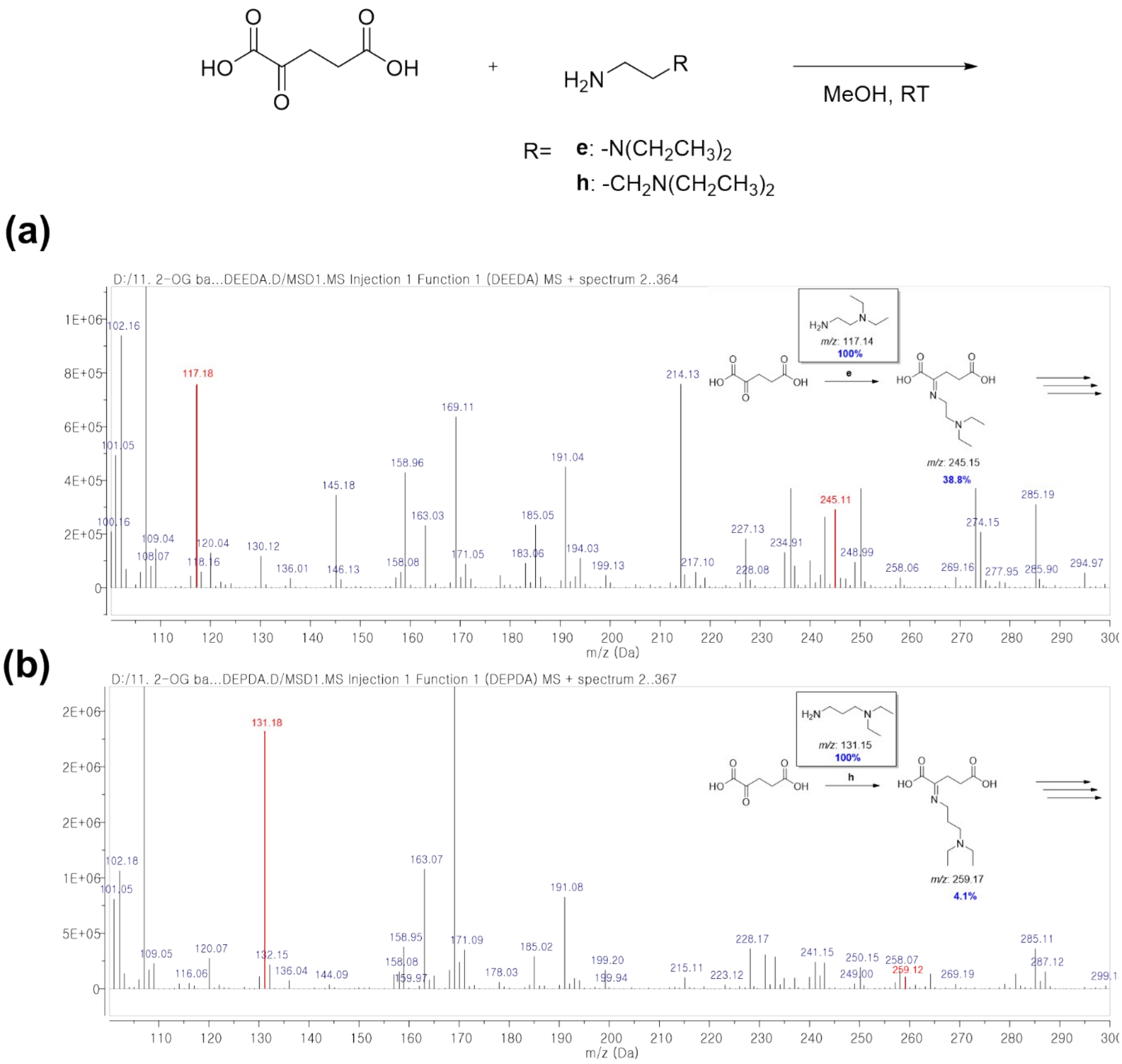

Figure S4. Difference of the imine intermediate formation from DEEDA (**e**) and *N,N*-diethylpropylenediamine (**h**). The imine product was produced much more effectively from DEEDA (m/z = 245) (a) than **h** (m/z = 259) (b).

(a)

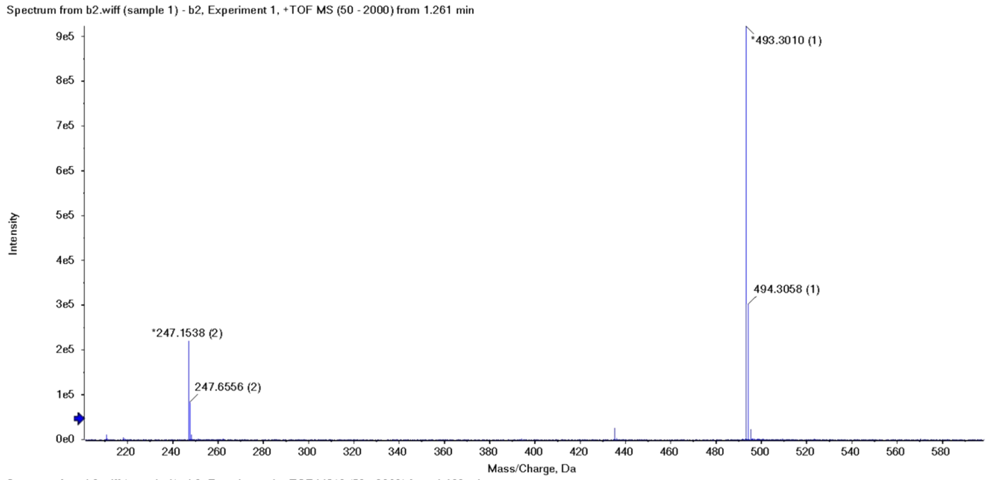

(b)

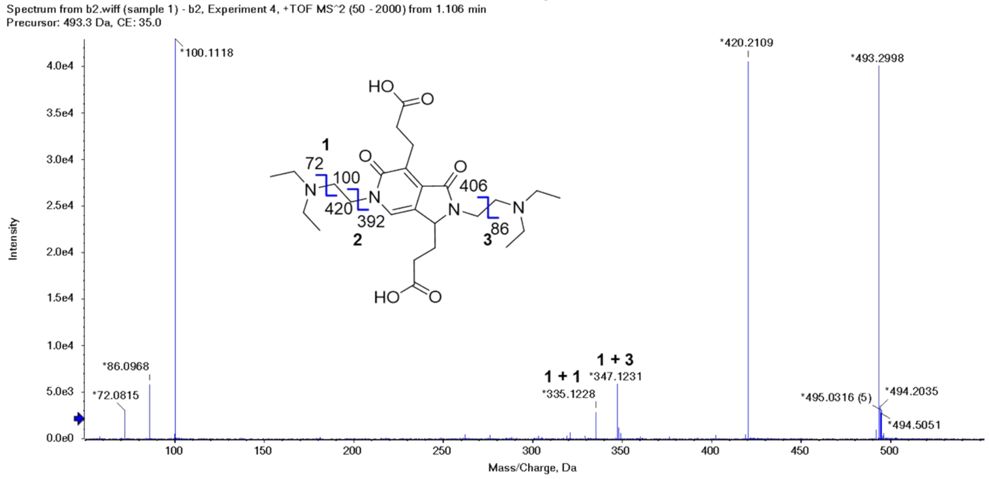

(c)

Formula	m/z^a	ppm^b	MS Rank^c	MSMS ppm^d	MSMS Rank^e
$C_{25}H_{40}N_4O_6$	493.3021	-0.9	1	4.1 (6)	1
$C_{32}H_{36}N_4O$	493.2962	11	7	7.1 (4)	2
$C_{33}H_{39}N_3O$	493.3088	-14.5	9	7.0 (3)	3
$C_{27}H_{43}NO_7$	493.3034	-3.7	3	2.6 (2)	4

a Experimentally detected m/z ratio of $[M + H]^+$; b m/z ratio difference (in units of ppm) between experimentally detected and theoretical *m/z* ratio of a given ion; c Rank-ordered agreement between experimental and predicted MS of a given formula. d average *m/z* ratio difference (in units of ppm) between experimentally detected and theoretical *m/z* ratio of given fragments ion (number of matched m/z of fragments to a given formula was assigned in bracket); e Rank-ordered agreement between experimental and predicted MSMS of a given formula.

Figure S5. HRLC-MS (a) and MS-MS (b) of the compound **1** (DTPP). (c) Table of the molecular formulas predicted by the post-processing software based on the *m/z* value (493.3010) and the fragmentation property.

5. DTPP-based fluorometric analysis

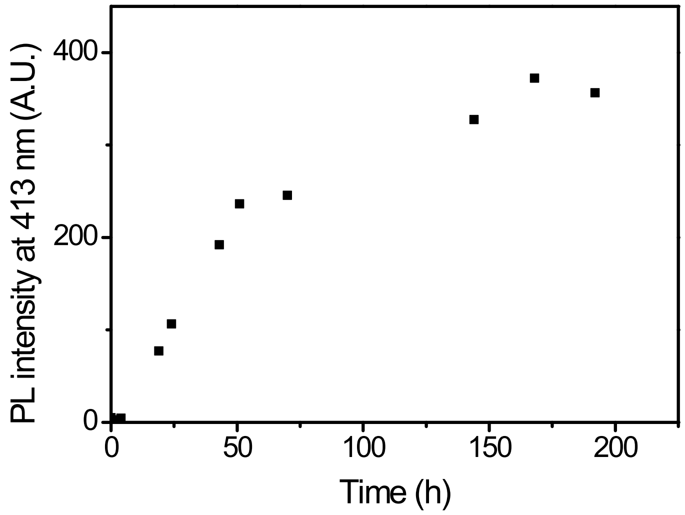

Figure S6. The long-term time-course of the fluorescence generation at 413 nm (λ_{ex} = 343 nm) of a 2-OG/DEEDA mixture (10 mM in MeOH).

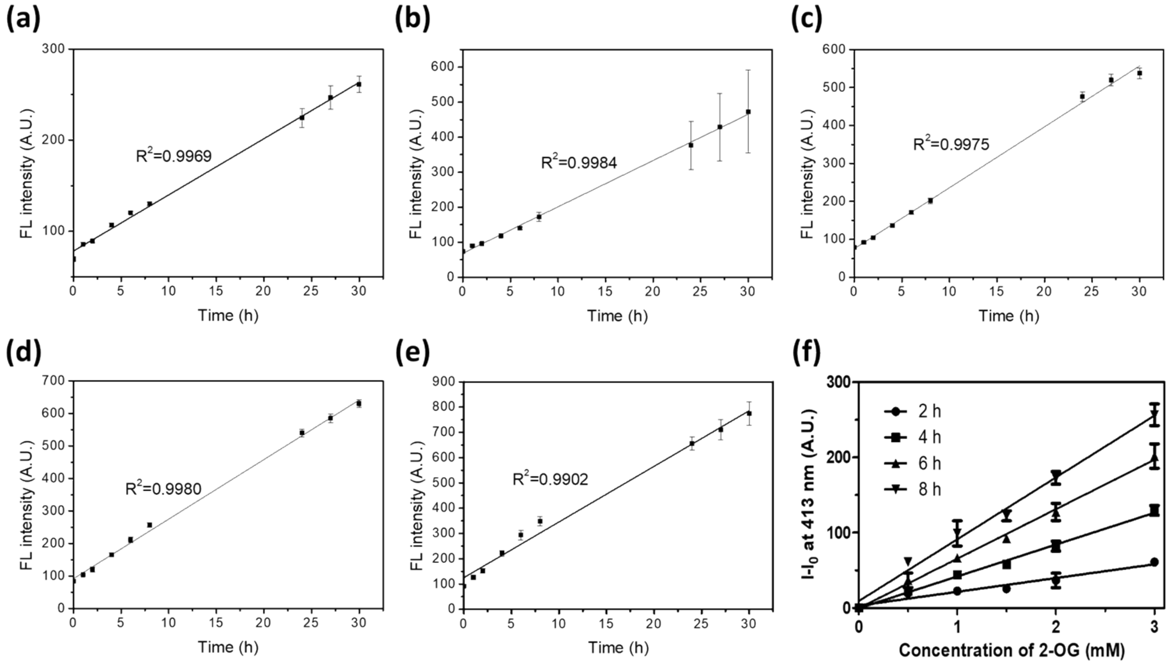

Figure S7. Time-dependent fluorescent generation in the mixture of a 10 mM DEEDA and 0.5 mM (a), 1.0 mM (b), 1.5 mM (c), 2.0 mM (d), and 3.0 mM (e) of 2-OG in methanol after incubation for 24 h at room temperature. (f) Fluorescence intensity-2-OG concentration relationship of the mixture at each time point. The DEEDA concentration was fixed as 10 mM. The intensity was measured at 413 nm (λ_{ex} = 343 nm). Data are means (± S.D.).

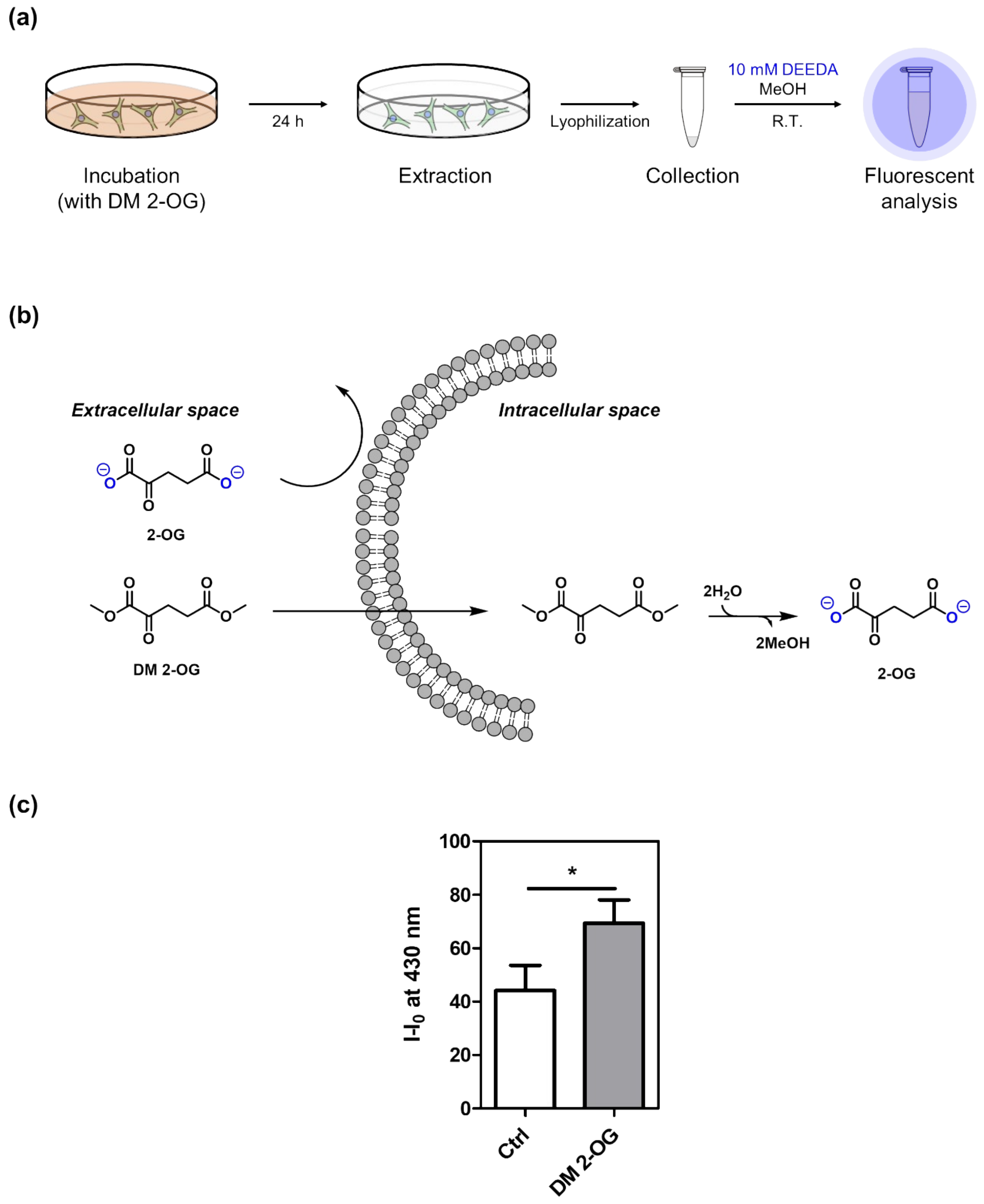

Figure S8. DTPP-based fluorometric analysis of 2-OG in cells. (a) Schematic diagram of the fluorometric analysis. The intracellular concentration of 2-OG was enhanced by pre-treatment of DM 2-OG (3.75 mM) for 24 h. The cell extracts were analyzed by the DTPP-based fluorometric analysis using DEEDA. (b) Enhanced permeation of DM 2-OG into cells and successive hydrolysis into 2-OG. (c) The fluorescence intensity of the cell extracts after the development of the DTPP fluorescence for 24 h. Data are means (± S.D.). $^*p < 0.05$ as determined by Student's t-test.

6. NMR data

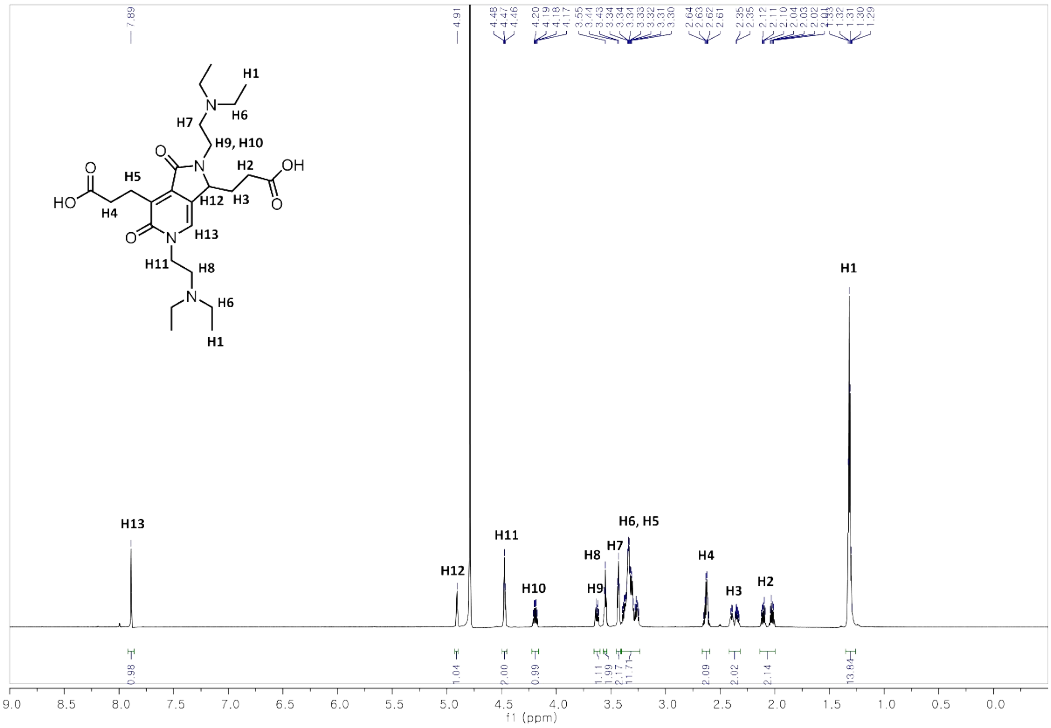

Figure S9. ^{1}H NMR of the compound **1** (DTPP) (D_2O, 850 MHz).

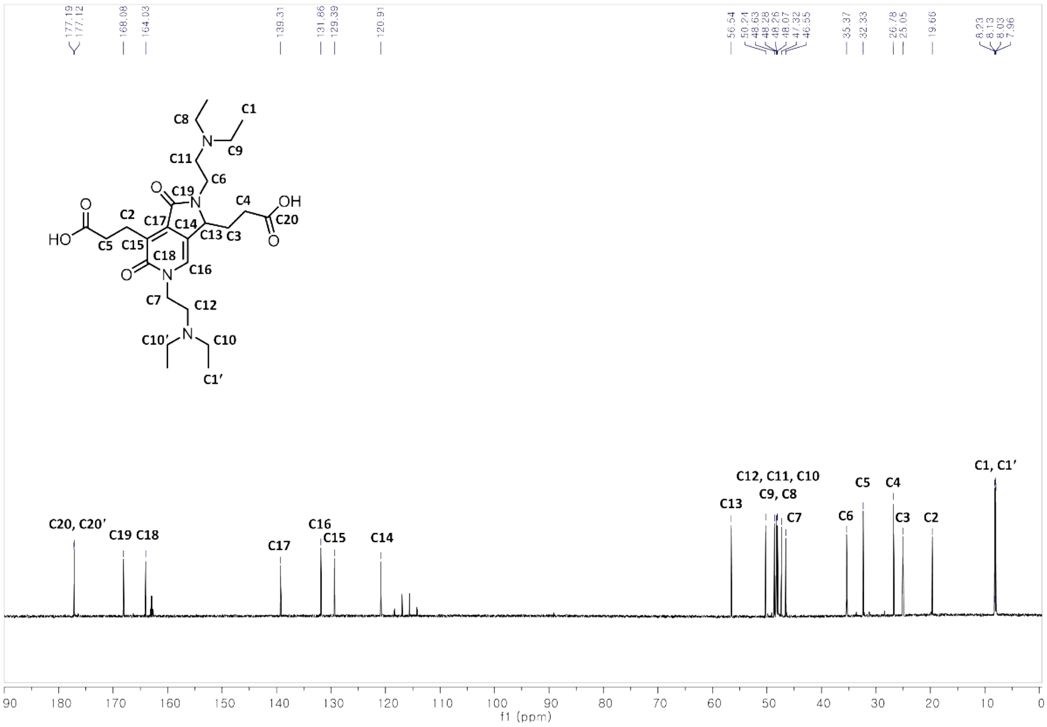

Figure S10. ^{13}C NMR of **1** (DTPP) (D_2O, 850 MHz).

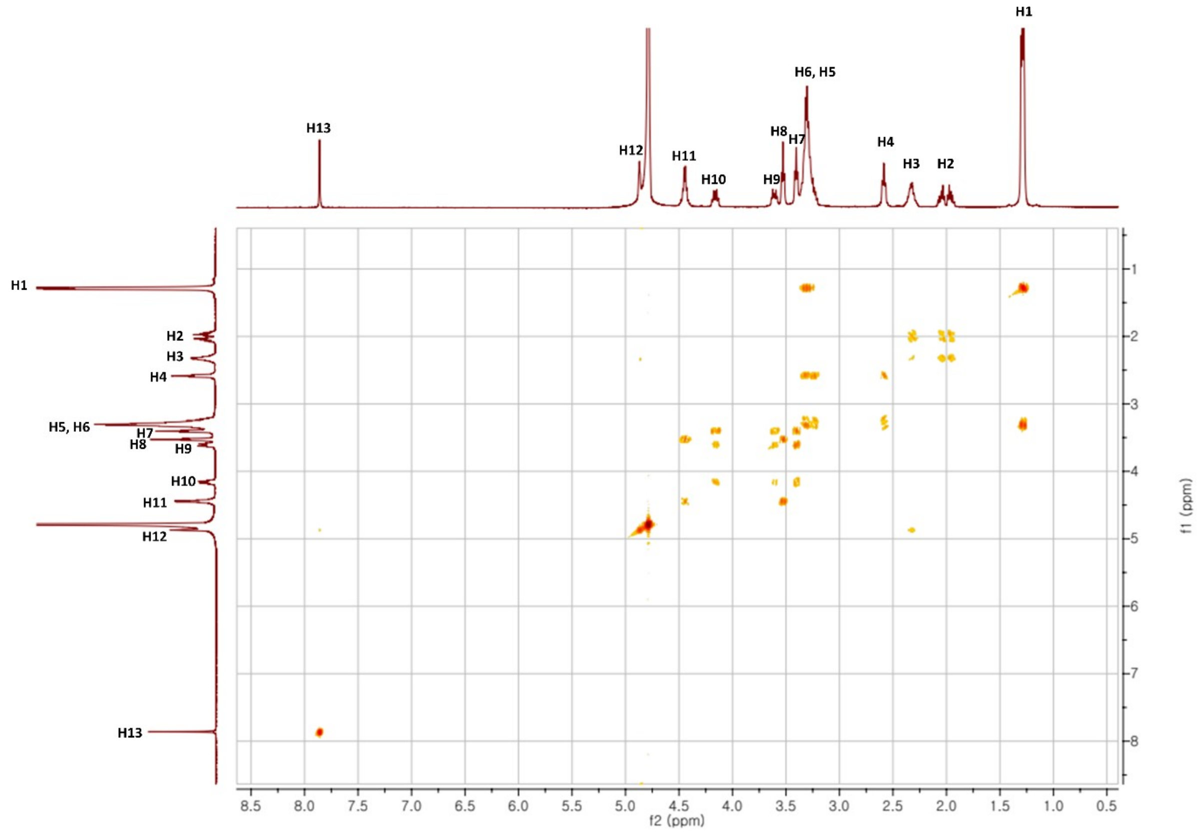

Figure S11. COSY of the compound **1** (DTPP) (D_2O, 500 MHz).

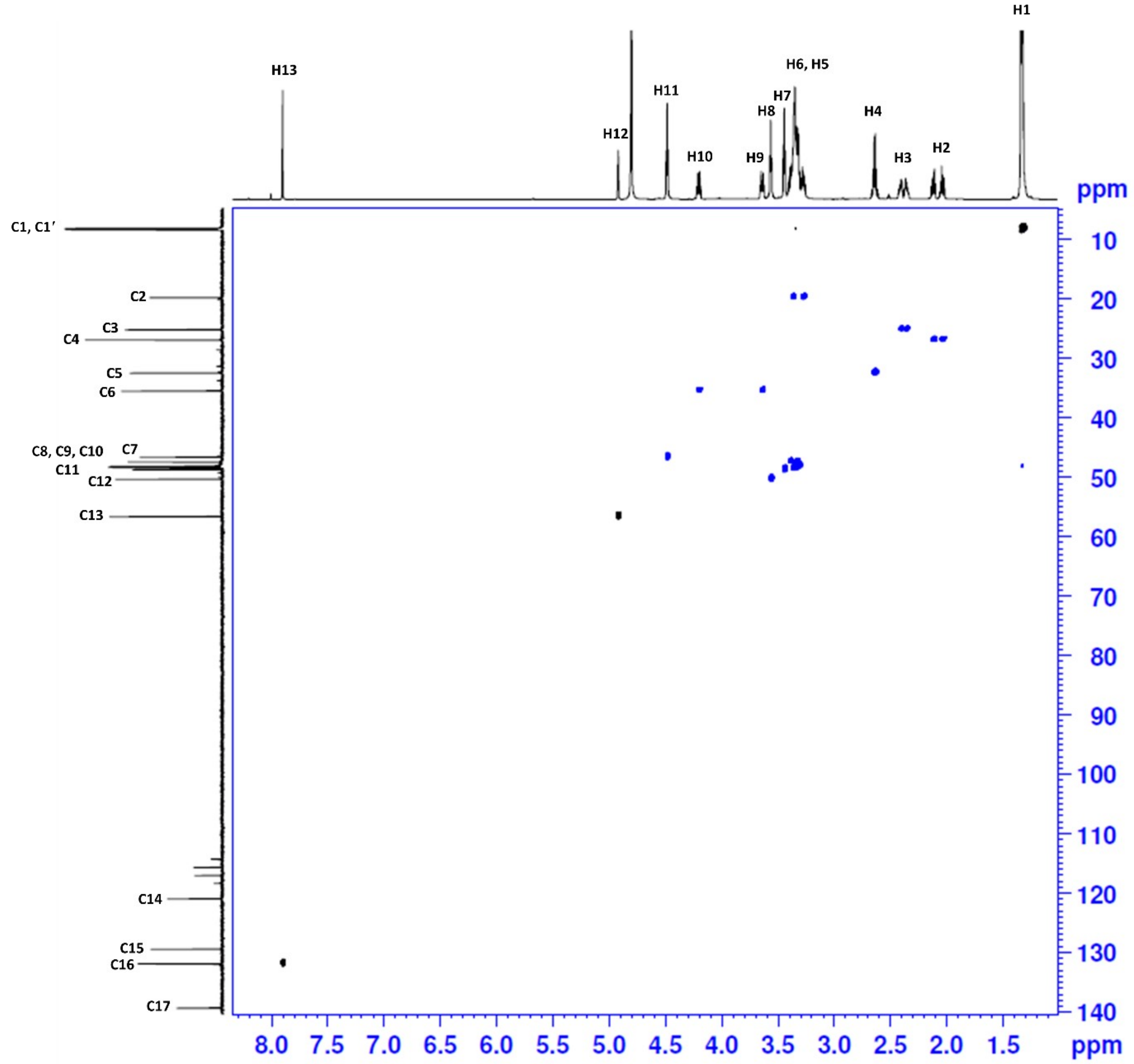

Figure S12. HSQC of the compound **1** (DTPP) (D_2O, 850 MHz).

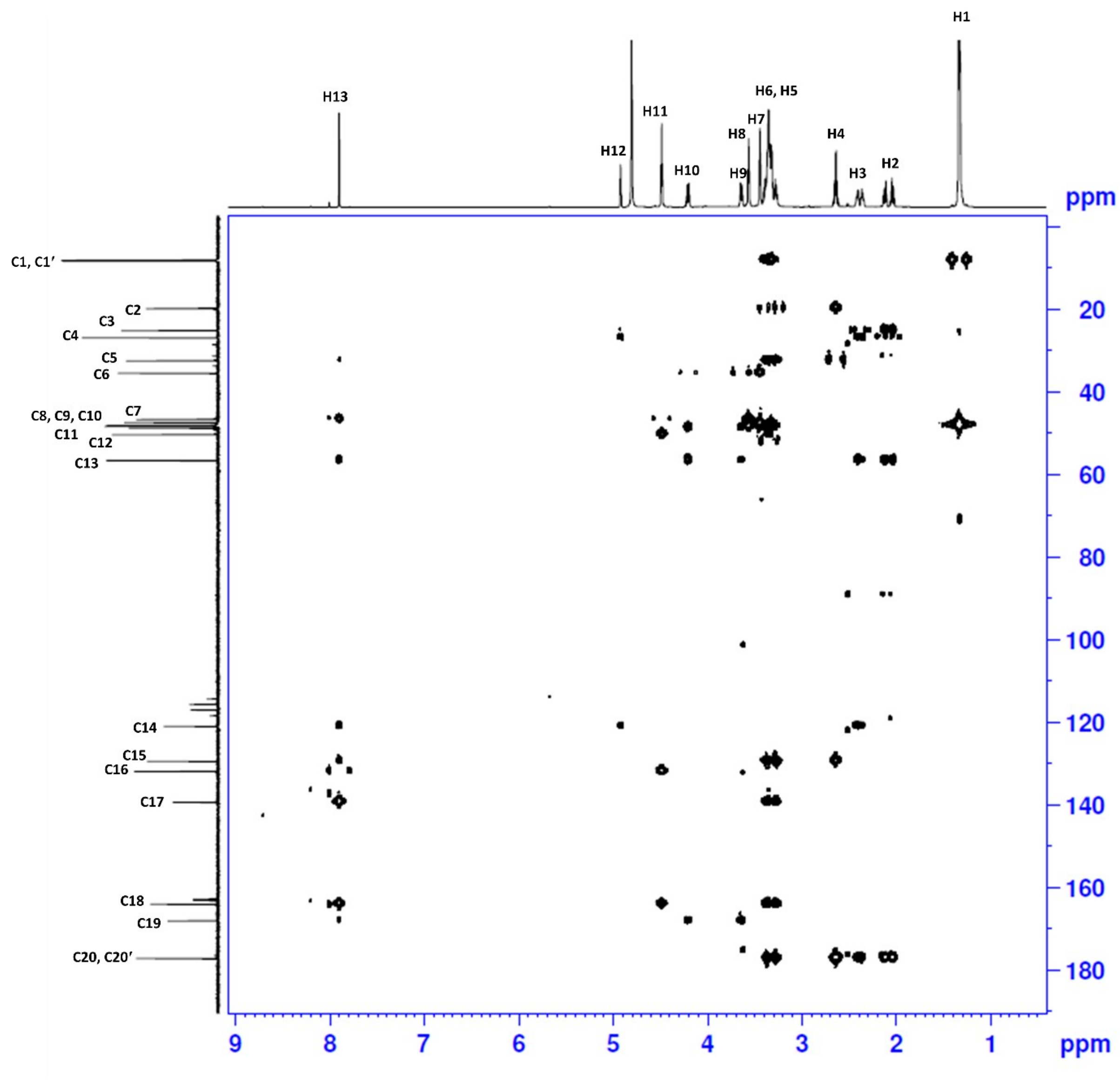

Figure S13. HMBC of the compound **1** (DTPP) (D_2O, 850 MHz).

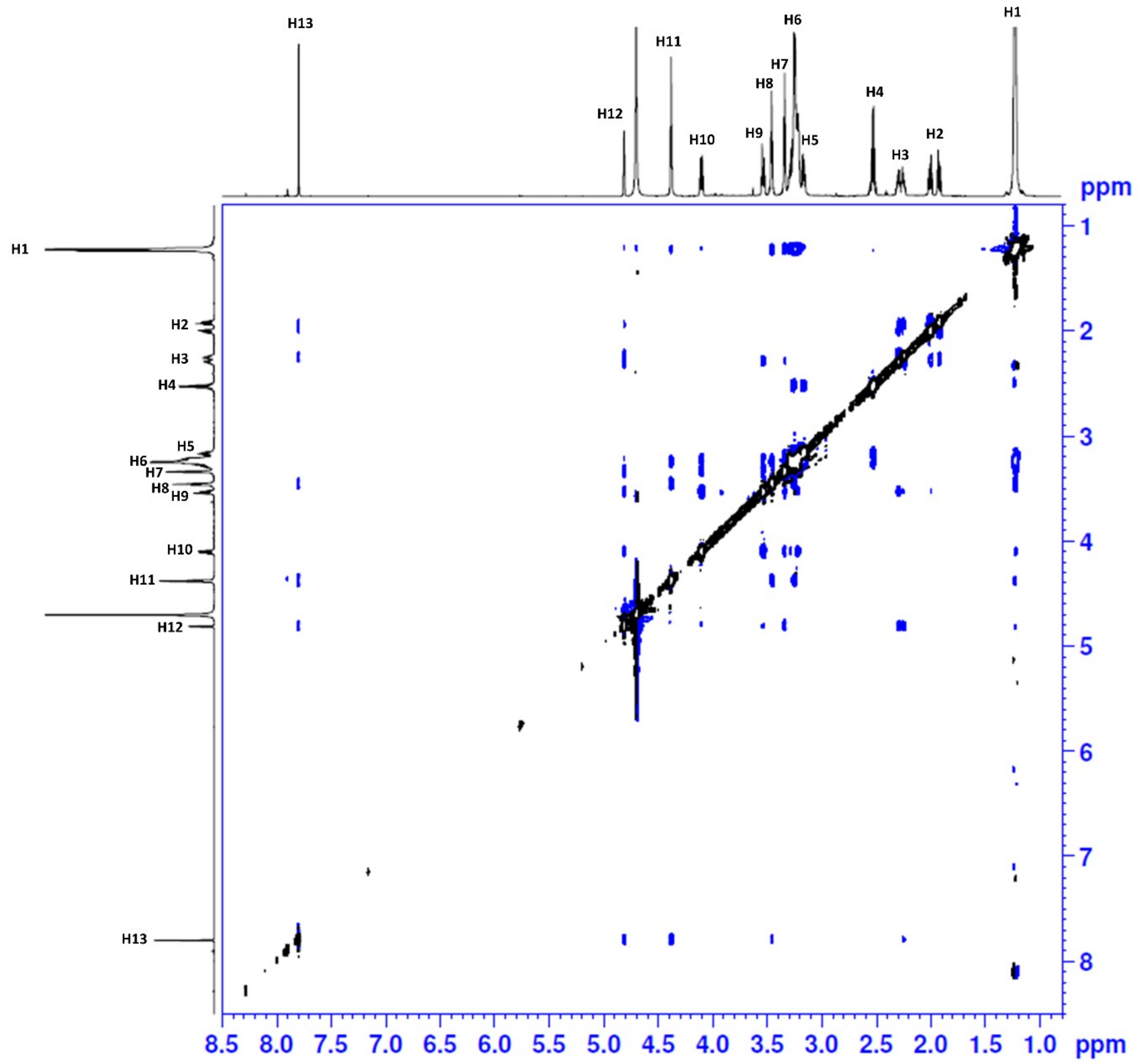

Figure S14. NOESY of the compound **1** (DTPP) (D_2O, 850 MHz).

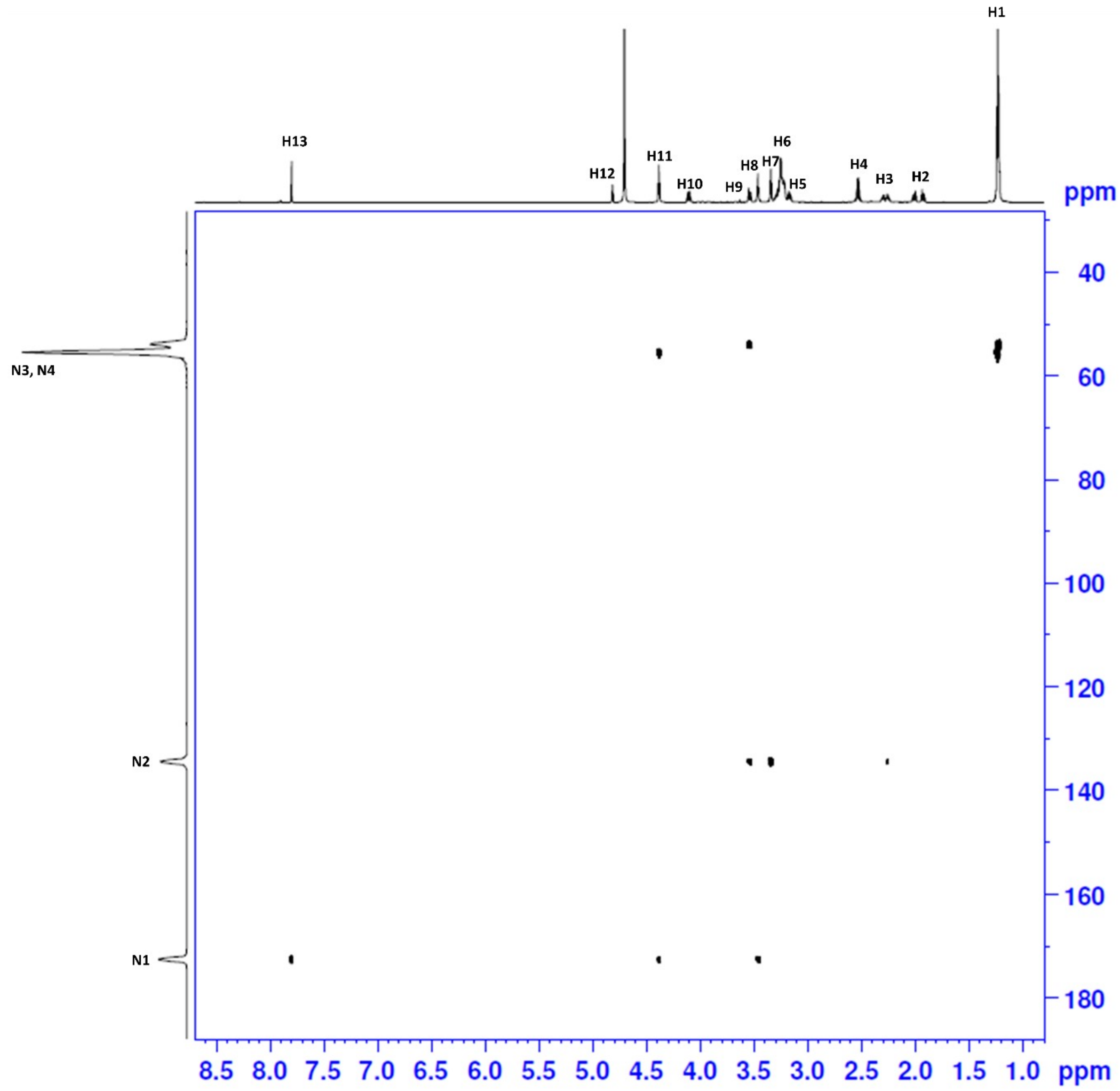

Figure S15. ^{1}H-^{15}N HMBC of the compound **1** (DTPP) (D_2O, 850 MHz).

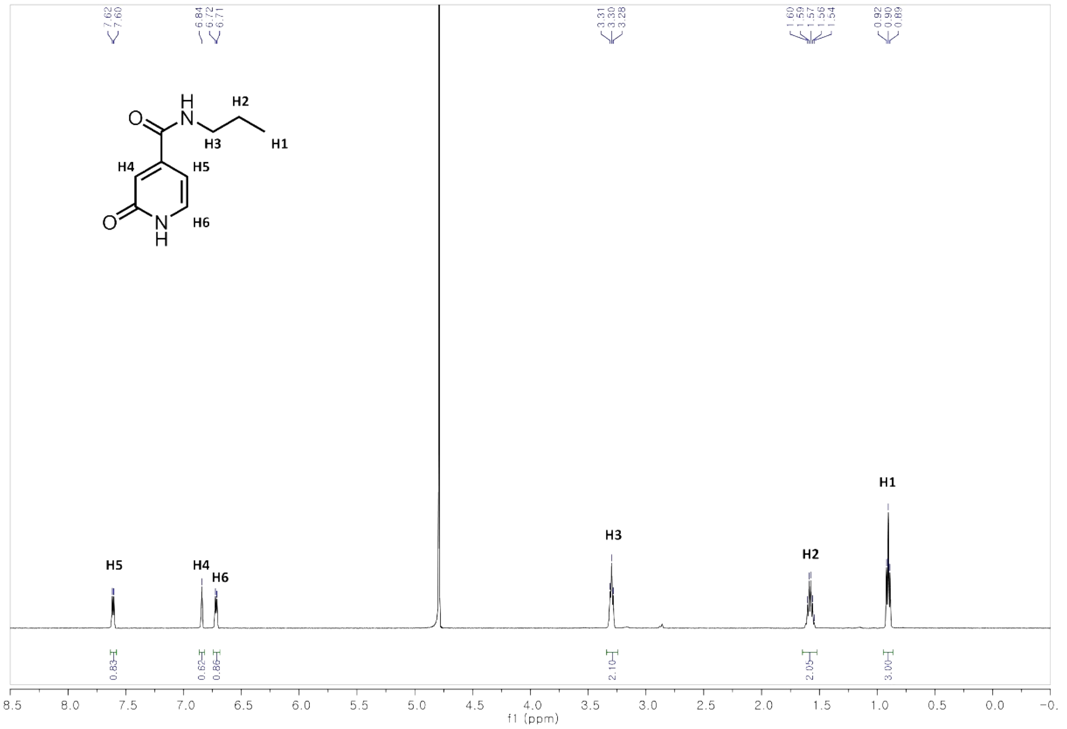

Figure S16. ^{1}H NMR of the compound **4** (D_2O, 500 MHz).

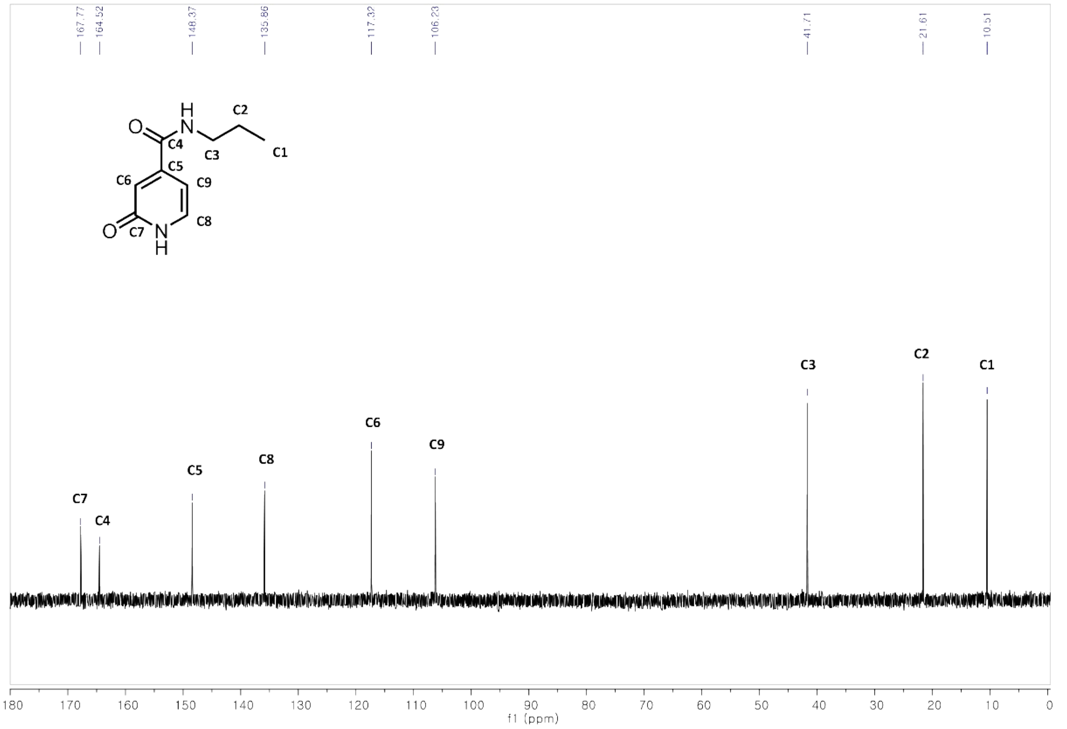

Figure S17. ^{13}C NMR of the compound **4** (D_2O, 500 MHz).

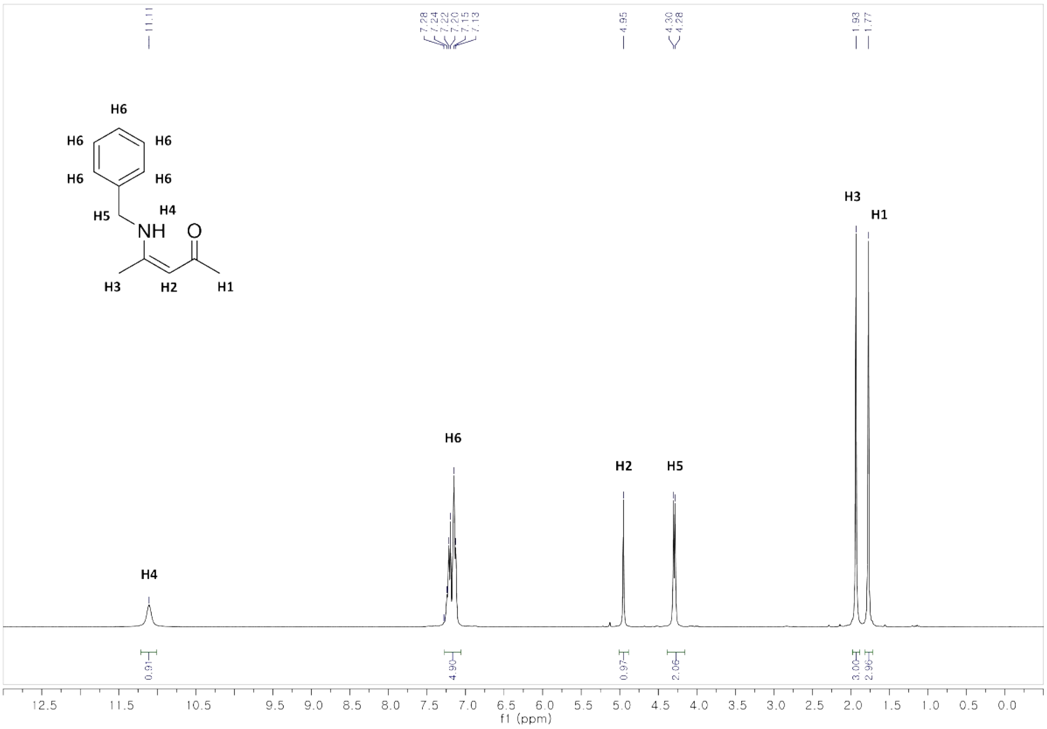

Figure S18. ^{1}H NMR of **2-(Benzylamino)prop-l-enyl methyl ketone** ($CDCl_3$, 500 MHz).

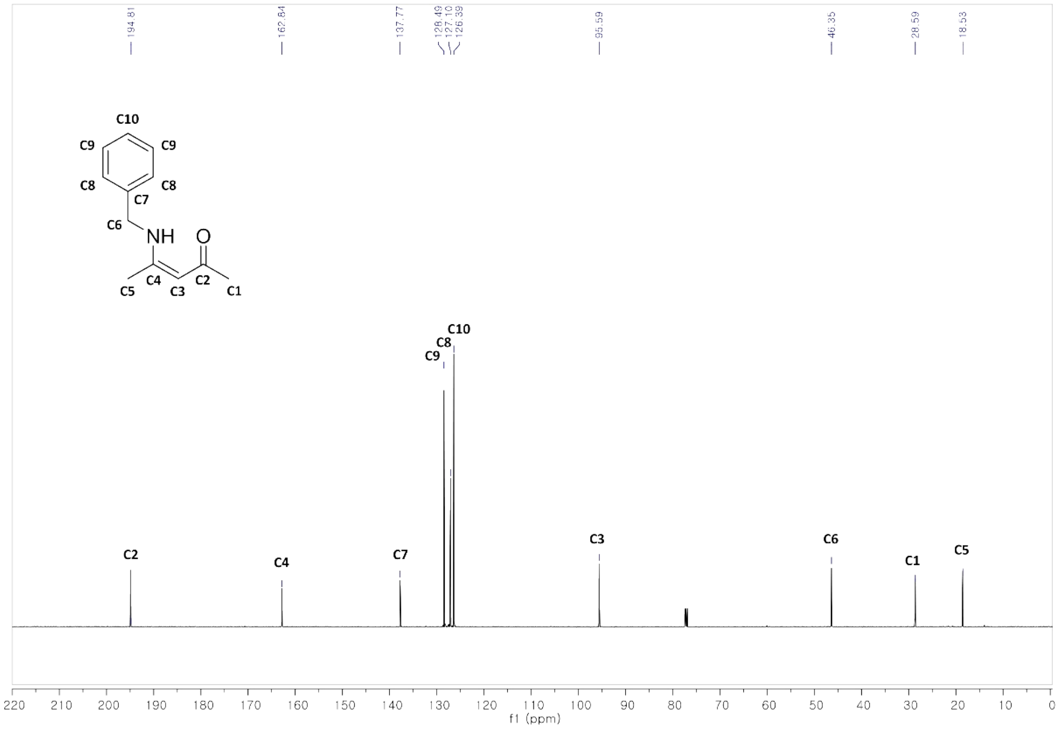

Figure S19. ^{13}C NMR of **2-(Benzylamino)prop-l-enyl methyl ketone** ($CDCl_3$, 500 MHz).

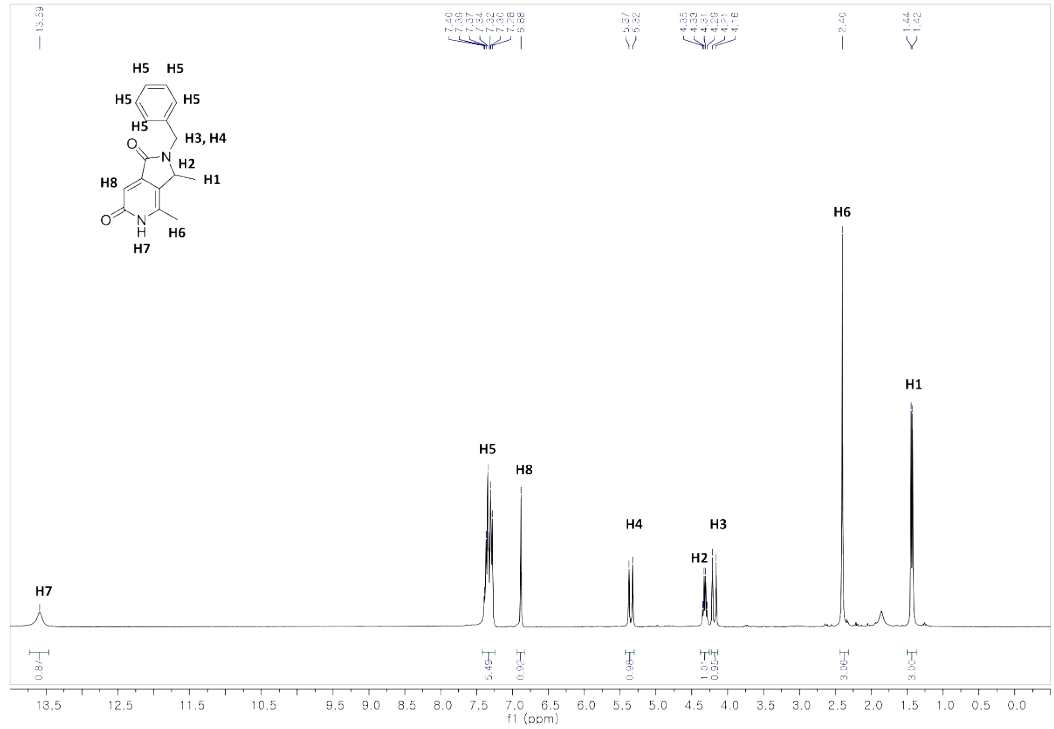

Figure S20. ^{1}H NMR of the compound **5** ($CDCl_3$, 500 MHz).

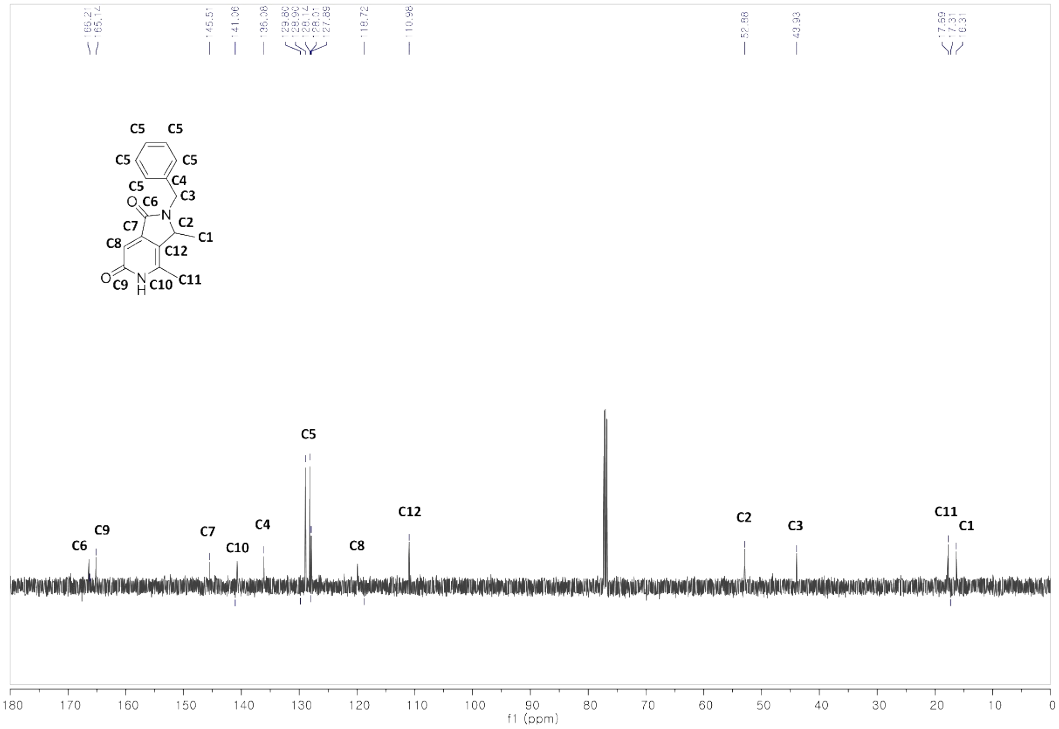

Figure S21. ^{13}C NMR of the compound **5** ($CDCl_3$, 500 MHz).

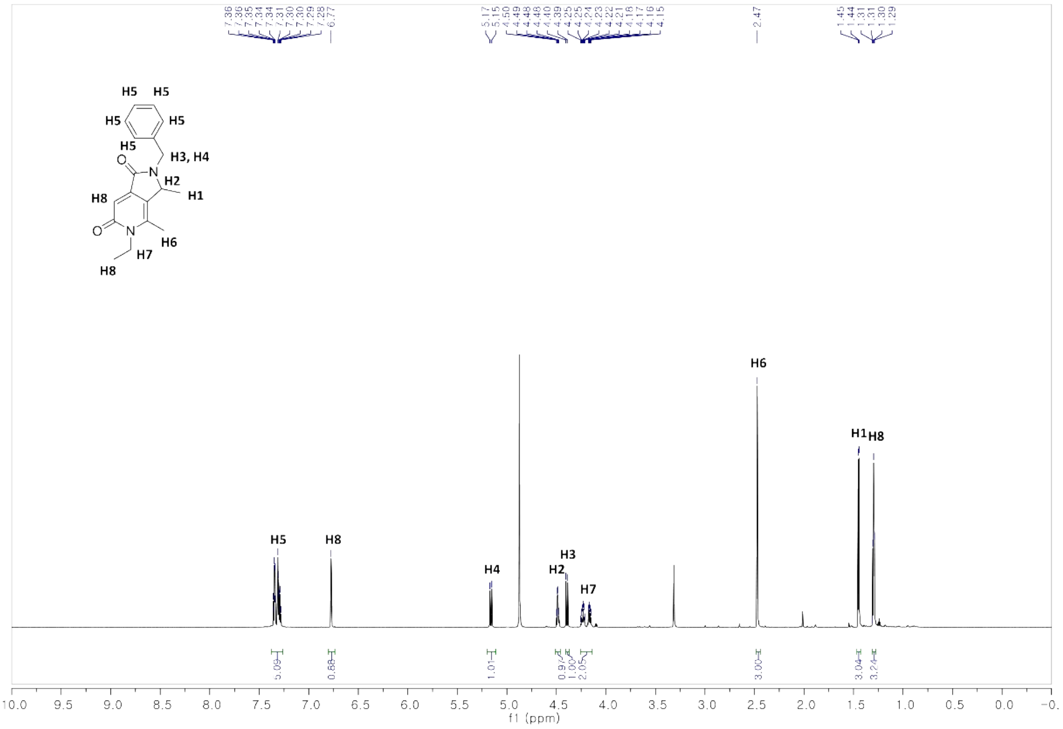

Figure S22. ^{1}H NMR of the compound **6** (MeOD, 850 MHz).

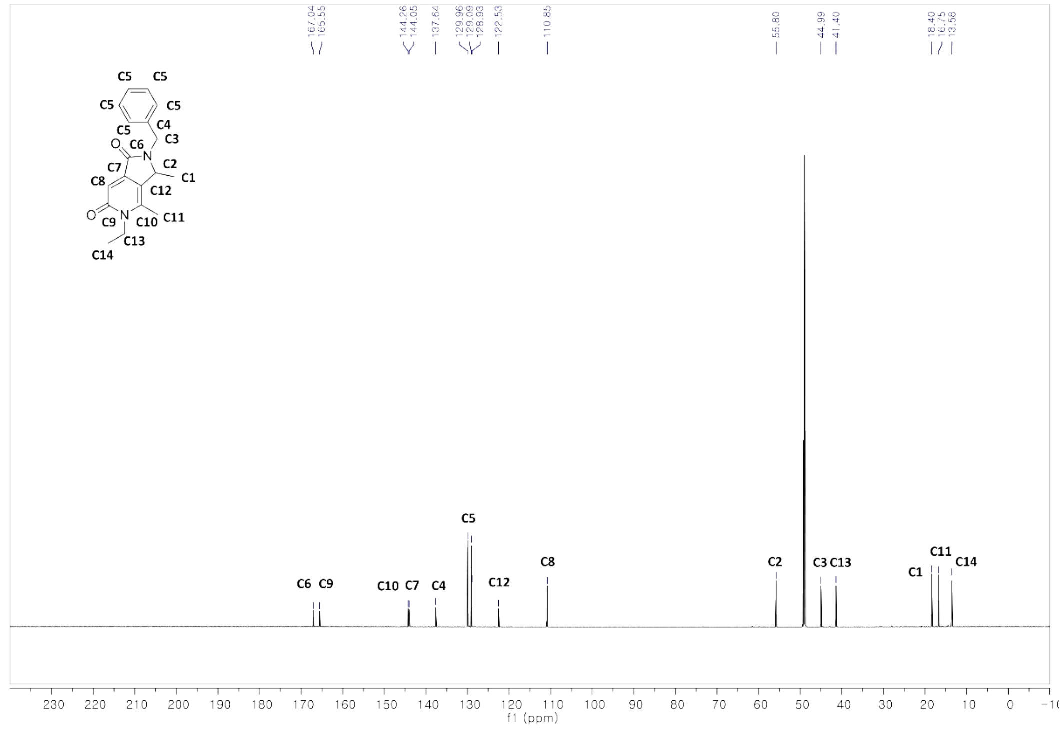

Figure S23. ^{13}C NMR of the compound **6** (MeOD, 850 MHz).

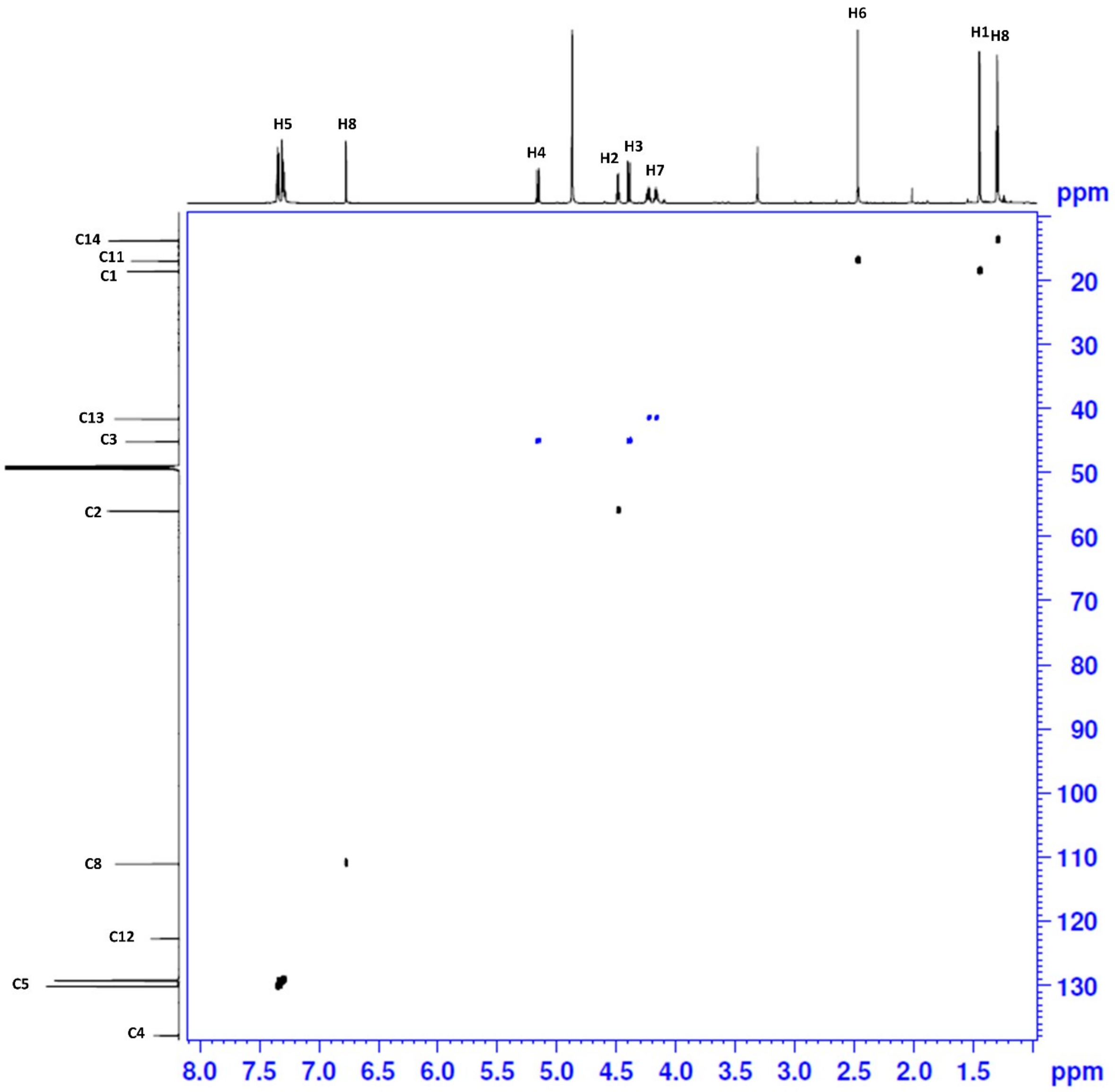

Figure S24. HSQC of the compound **6** ($CDCl_3$, 850 MHz).

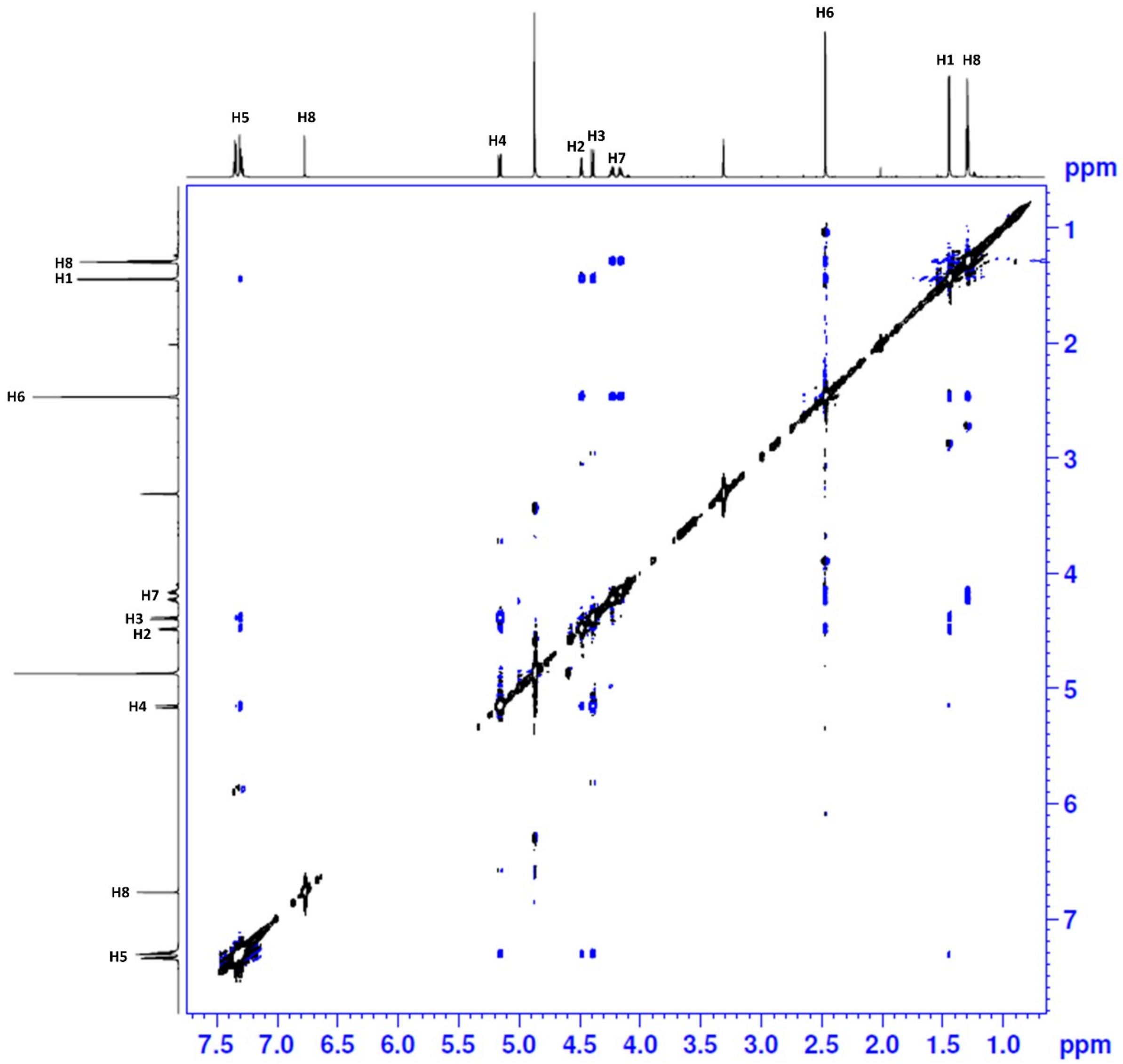

Figure S25. NOESY of the compound **6** ($CDCl_3$, 850 MHz).

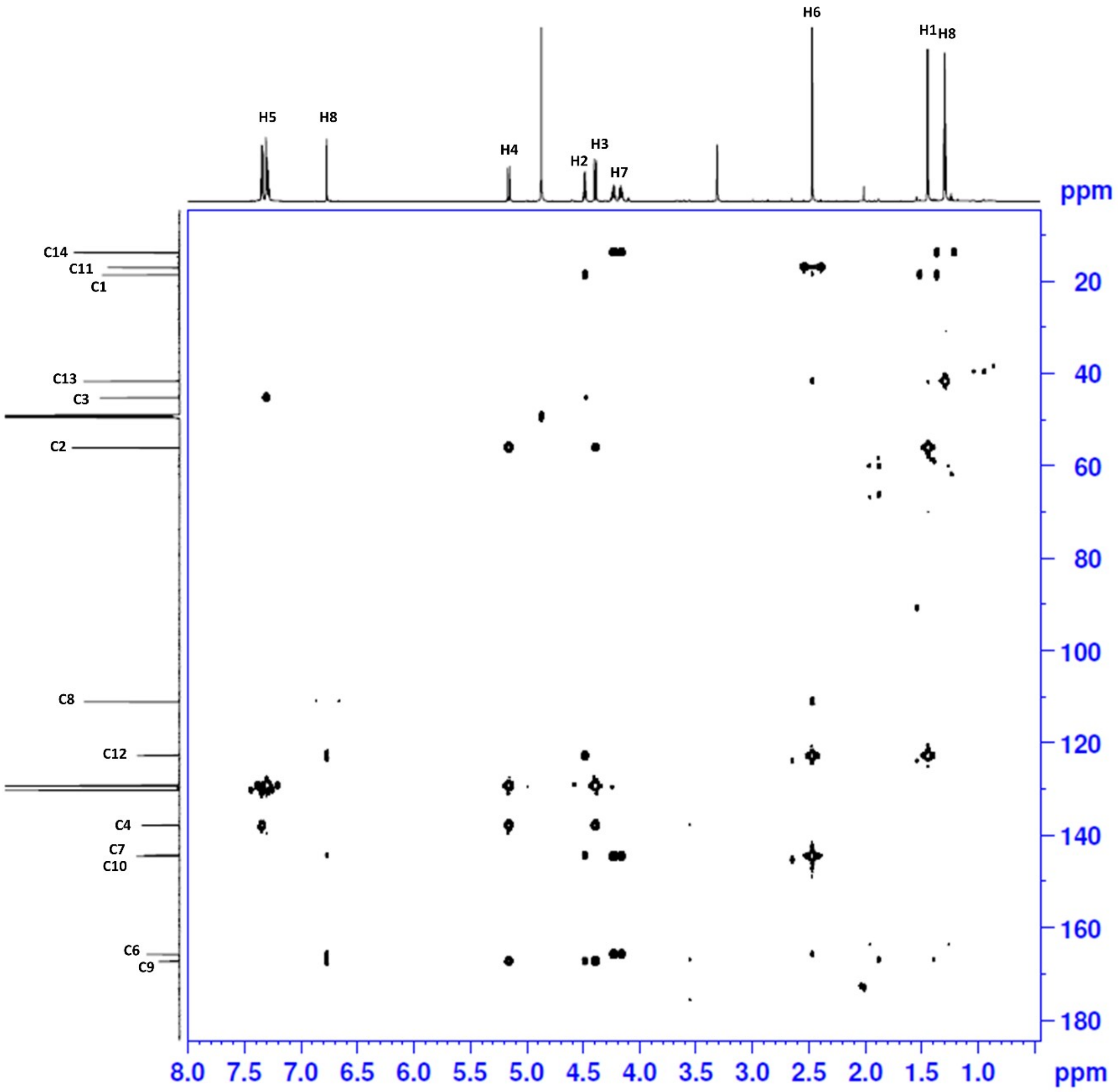

Figure S26. HMBC of the compound **6** ($CDCl_3$, 850 MHz).

7. SC-XRD analysis

Crystallization of 6.

Because DTPP is difficult to be crystallized, the compound **6** with the same core structure was crystallized instead. The NMR patterns of compound **6** (Figure S19-S23) are nearly identical to those of the DTPP core structure. Vapor diffusion method was used to crystallize **6**. In an inner vial, 5.4 mg of **6** was dissolved in 0.5 mL chloroform. In an outer vial, 2 mL of cyclohexane was added and sealed with paraffin film. After 24 h-incubation at room temperature, crystal was obtained.

X-ray crystallographic analysis

A suitable crystal was selected and recorded on a SuperNova, Dual, Cu at home/near, AtlasS2 diffractometer. The crystal was kept at 292.6(3) K during the data collection. Using Olex2 [2], the structure was solved with the ShelXT [3] structure solution program using Intrinsic Phasing and refined with the ShelXL [4] refinement package using Least Squares minimization.

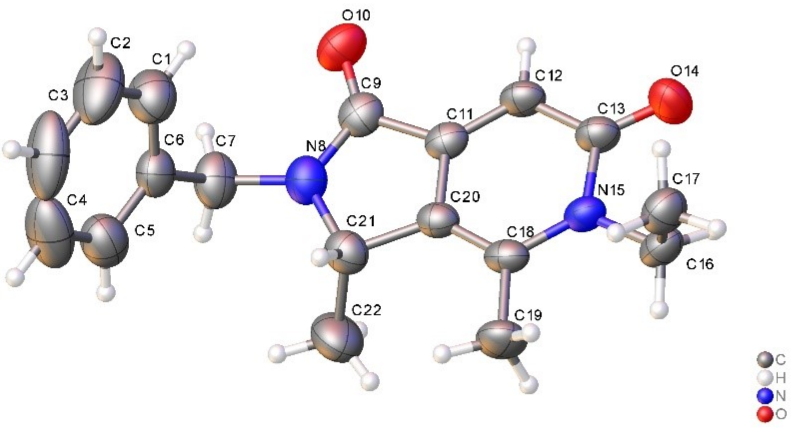
O10
C2
C1
C3
C6
C7
N8
C9
C11
C12
O14
C13
C4
C5
C21
C20
N15
C17
C18
C16
C22
C19
C
H
N
O

```
Bond precision:   C-C = 0.0037 A          Wavelength=0.71073

Cell:              a=11.6320(8)       b=9.0024(9)      c=29.319(2)
                   alpha=90           beta=90          gamma=90
Temperature:       293 K

                  Calculated                 Reported
Volume            3070.2(4)                  3070.2(4)
Space group       P b c a                    P b c a
Hall group        -P 2ac 2ab                 -P 2ac 2ab
Moiety formula    C18 H20 N2 O2              C18 H20 N2 O2
Sum formula       C18 H20 N2 O2              C18 H20 N2 O2
Mr                296.36                     296.36
Dx,g cm-3         1.282                      1.282
Z                 8                          8
Mu (mm-1)         0.084                      0.084
F000              1264.0                     1264.0
F000'             1264.52
h,k,lmax          16,12,40                   14,12,37
Nref              4301                       3770
Tmin,Tmax         0.990,0.994                0.523,1.000
Tmin'             0.979

Correction method= # Reported T Limits: Tmin=0.523 Tmax=1.000
AbsCorr = MULTI-SCAN

Data completeness= 0.877           Theta(max)= 29.535

R(reflections)= 0.0638( 2106)      wR2(reflections)= 0.1553( 3770)

S = 1.035                  Npar= 202
```

Figure S27. The X-ray crystal structure and structure refinement for compound **6**

8. References

[1] Shah, K. R., & Blanton Jr, C. D. Reaction of maleimides and ethyl 3-aminocrotonates. A reinvestiation leading to an improved synthesis of pyrrolo [3, 4-c] pyridines. *J. Org. Chem.* **1982**, *47*, 502-508.

[2] Dolomanov, O. V., Bourhis, L. J., Gildea, R. J., Howard, J. A., & Puschmann, H. OLEX2: a complete structure solution, refinement and analysis program. *J. Appl. Crystallogr.* **2009**, *42*, 339-341.

[3] Sheldrick, G. M. SHELXT - integrated space-group and crystal-structure determination *Acta Cryst.* **2015**, *A71*, 3–8.

[4] Sheldrick, G. M. Crystal structure refinement with SHELXL. *Acta Cryst*. **2015**, *C71*, 3–8.
